# Influence of body mass index on health complains and life satisfaction

**DOI:** 10.1007/s11136-023-03557-0

**Published:** 2023-12-01

**Authors:** Claudia Prieto-Latorre, Luis Alejandro Lopez-Agudo, Oscar David Marcenaro-Gutierrez

**Affiliations:** Departamento de Economía Aplicada (Estadística y Econometría), Facultad de Ciencias Económicas y Empresariales, Universidad de Málaga, Plaza de El Ejido s/n, 29013 Málaga, Spain

**Keywords:** Body mass index, Instrumental variable, Psychosomatic complaints, Life satisfaction, Bullying, I20, I31

## Abstract

**Purpose:**

This research work investigates the influence of children’s weight status on well-being and school context in a sample of Spanish adolescences.

**Methods:**

The Spanish records from the 2013–14 Health Behaviour in School-Aged Children Survey are used, which gathers 9,565 adolescences aged 11, 13 and 15. Studies do not usually address the endogeneity of body mass index when analysing their effect on life satisfaction and health complaints, thus resulting in biased estimates. Considering the endogeneity of body mass index, we use the frequency of alcohol consumption as an instrumental variable in order to obtain consistent estimates of its influence.

**Results:**

The two-stage least squares estimation shows that children’s body mass index has a significant negative influence on health complaints and it conditions the way children relate to each other at school. Likewise, results report significant influence on children’s subjective well-being and their self-assessment of general health.

**Conclusions:**

The results of this study provide compelling evidence that BMI plays a crucial role in shaping adolescents’ well-being and their interactions with peers at school. These findings underscore the importance of addressing childhood overweight and promoting healthy body mass index levels. Furthermore, the study highlights the need for targeted policy interventions to combat the social stigma associated with being overweight, fostering a more inclusive and supportive school environment for all students.

**Supplementary Information:**

The online version contains supplementary material available at 10.1007/s11136-023-03557-0.

## Introduction

Beyond health concerns, maintaining an optimal weight according to one’s height is crucial for both cognitive and non-cognitive development, as well as for various aspects of life, including social relationships [1, 2]. This is important through life, and it becomes particularly prominent during adolescence, a period marked by strong stigmatization of heavier body sizes and its impact on social acceptance and friendships [3]. This study aims to explore the influence of body mass index (BMI) on health complaints and psychosocial relationships of Spanish teenagers following a causal methodological approach.

Children with excess weight experience poorer physical health. In this regard, childhood obesity is associated with the development of a number of medical complications, such as type 2 diabetes, hypertension, sleep apnea or cholesterol disorders [4]. All these health issues manifest as musculoskeletal, neurological and gastrointestinal pains [5] and lead to higher healthcare expenses compared to children with a healthy weight [6].

In addition to adverse effects on health status, being overweight is associated with lower life satisfaction [7]. Firstly, somatic complaints reduce the quality of life of children who are overweight [8]. Secondly, overweight children are also more likely to suffer from depression and anxiety disorders [9], which are also negatively associated with life satisfaction [10]. A third channel mediating the relationship between life satisfaction and overweight is social bias. Research indicates that overweight children are more frequently targets of bullying due to factors like social marginalization, lower self-esteem and body dissatisfaction, among others [11–14]. In fact, adolescents themselves perceive weight status as one of the main reasons to be bullied by their peers [15], which underscores the prevalence of weight-based stigma among teenagers [16]. This discomfort in the learning environment may explain why overweight children are more likely to be absent from school and tend to have a lower academic performance compared to their normal-weight peers [17–19]. Due to the associated risks of excess weight and its escalating prevalence, childhood overweight and obesity are considered as a worldwide epidemic in modern society [20].

The percentage of children with excess weight is particularly alarming in Spain, where 40.6% of children^1^ fall into the overweight or obese category. This figure surpassed that of neighbouring countries, with rates of 16.5% in France, 29.6% in Portugal and 29.8% in Italy. Specifically, Spain is ranked third among the European countries with more overweight children [21]. This is surprising, given that Spain encompasses the Mediterranean diet, argued as one of the healthiest [22]. In this context, it is especially interesting to deepen the understanding of the consequences of childhood weight on perceived health (psychosomatic complaints) and school life in Spain. Therefore, our first aim is to provide new evidence on this issue. Specifically, we use the data from the “Health Behaviour in School-aged Children” for Spain—2014 (the last survey publicly available); using this dataset, we investigate how body mass index conditions students’ perceived health and school life, including violence, bullying issues and peer support.

Yet, despite the widespread interest in the consequences of weight status, relatively few works have tried to mitigate the endogenous part of weight status [23]. In this regard, children’s weight status can be endogenous since it is associated with socioeconomic conditions and individual’s surroundings [24] and these variables are determinants of some outcomes explored in this study, such as life satisfaction. Moreover, the dependencies explored herein could suffer from reverse causality problems. Let us give an example: Do children feel low because they are overweight, or could it be that feeling low predisposes them to gain weight? We found a limited number of studies which have tried to control the biases produced by such confounding problems of weight status on diverse outcomes. Previous research focusing on children's development have used the weight of a biological relative [25–28], genetic markers [28], child’s height [29] and past weight status of child [28, 30] as instrument for child's weight.

In light of these methodological considerations, the evidence provided herein contributes to the existing literature in two significant ways. First, our research implements an instrumental variable procedure, using as instrument the frequency of alcohol consumption. While previous literature has frequently addressed the association between body mass index or overweight/obesity status, few studies have gone a step further and try to get close to a causal effect. The instrumental variable approach allows us to obtain consistent estimates of the influence of body mass index. Second, we provide new empirical evidence about the influence of body mass index on health complaints and school-related factors in Spain. Although some research studies have explored the consequences of weight status in the Spanish case (see [31, 32], to the best of our knowledge, there are no previous large-scale studies which have systematically investigated the link between psychosomatic complaints and the body mass index in this country.^2^

## Data

This research is based on the Spanish records from the 2013–14 Health Behaviour in School-Aged Children Survey (HBSC). The HBSC is a collaborative study coordinated and sponsored by the World Health Organization (WHO), carried out each 4 years. The goal is to collect information about adolescents’ health. According to the WHO, “health is a state of complete physical, mental and social well-being and not merely the absence of disease or infirmity”.^3^ Following this definition, they collect data related to physical health, well-being, and social environment at home and at school, food and diet, substance use and physical activity. The survey consisted of an online questionnaire completed by adolescences aged 11, 13 and 15 years old in the classroom setting. To obtain a representative sample of the Spanish population of these ages, participants were recruited using a multi-stage random sampling stratified by conglomerates, taking into account the age, the Autonomous Community and the ownership of the school (public or private) [34]. The Spanish data were collected between March and December 2014.

The number of students participating in Spain was 11,136. To be eligible for the present research, students must have answered the questions related to both height and weight. In particular, around 86% of the sample answer both questions; this left a total of 9,565 students for consideration in the current analysis. Table A1 (Appendix) shows the descriptive statistics of the variables employed in the research. We have analysed if there are any differences between the sample used and the sample excluded by performing a test of mean differences and we have identified significant differences in the higher frequency of feeling psychosomatic complaints, high levels of life satisfaction, high participation in fights and bullying outcomes, and in the set of variables which measure the socioeconomic status (Table A1, Appendix). This should be taken into account when interpreting the results. Besides that, missing answers in the dependent variables may reduce the sample.

Three sets of outcomes are explored in this study: subjective health complaints and life satisfaction, fighting and bullying, and peers’ support. Table A2 (Appendix) summarises the outcome variables—the exact wording of the questions can be seen at Table A1 (Appendix).

## Methodology

The main variable of interest in this research is the body mass index (BMI henceforth). This variable is a measure used to classify the population into severe thinness, thinness, normal weight, overweight and obesity, and it is derived from the relationship between height and weight:

$$BMI= \frac{weight (kg)}{{height}^{2} ({m}^{2})}$$

Both weight and height are self-reported. We intend to identify empirically the effect of body mass index on health and school outcomes. To begin, we estimate the following ordered probit model:

1 $${O}_{i}^{*}=\alpha +\beta {BMI}_{i}+\gamma {X}_{i}+\delta {S}_{i}+{u}_{i}$$

where ${O}_{i}^{*}$ is a latent outcome variable, further ${O}_{i}^{*}=k$ if ${d}_{k-1}<{O}_{i}^{*}<{d}_{k}$. The different outcomes are:

- Psychosocial complaints: headache, stomach-ache, backache, feeling nervous, difficulties in sleeping and feeling dizzy, with $k=\mathrm{0,1},\mathrm{2,3},4$ (0 = rarely or never, 1 = about every month, 2 = about every week, 3 = more than once/week, 4 = about every day).
- Self-rated general health with $k=\mathrm{0,1},\mathrm{2,3}$ (0 = poor, 1 = fair, 2 = good, 3 = excellent).
- Subjective life satisfaction with $k= \mathrm{0,1},\mathrm{2,3},\dots , 10$ (0 = worst possible life, 10 = best possible life).
- Times of physical fight with $k=0, 1, 2, \mathrm{3,4}$ (0 = none, 1 = 1 time, 2 = 2 times, 3 = 3 times, 4 = 4 times or more).
- Bullied others, been bullied, bullying others, cyberbullied by messages, cyberbullied by pictures with $k=\mathrm{0,1},\mathrm{2,3},4$ (0 = haven’t, 1 = once or twice, 2 = 2–3 times per month, 3 = once a week, 4 = several times a week).
- Friends try to help, can count on friends, have friends to share joys and sorrows, can talk about problems with friends with $k=\mathrm{1,2},3,\dots ,7$ (1 = very strongly disagree; 7 = very strongly agree).

The explanatory variables can be classified as:

- The study-specific variable is the body mass index, represented by BMI.
- Demographic characteristics. The vector of covariates $({X}_{i})$ comprises the students’ demographic characteristics (sex and immigrant status).
- Additional control variables. ${S}_{i}$ is a vector with detailed information about the socioeconomic and cultural status: parental occupation (classified from low to high), household possessions (family car, own bedrooms, number of computers, number of bathrooms, dishwasher in home, family holidays), students’ perceptions of family well-off and the practice of physical activity (frequency of doing vigorous physical activity and the hours of exercise per week).

A summary of the set of control variables can be found in Table A3 (Appendix). Since there are observations with missing socioeconomic and cultural characteristics, we replace those missing values with “0” and we add an indicator variable (i.e. missing flag variable). By doing so, we can maximize the number of observations in the sample; ${u}_{i}$ is the idiosyncratic error term.

The estimated $\beta$ coefficient captures the influence of one point increase of student’s BMI on the outcome of interest, controlling for student’s characteristics. However, this estimation may suffer from bias due to the unobserved factors that may be correlated with BMI and the outcomes under studied. For instance, greater fast food intakes, which lead to gain weight, have been found to be associated with lower life satisfaction [35]. Besides that, around 50% of body mass index variation is due to individual choices and environment, which suggests that BMI is not exogenous [36]. To solve this, we employ the instrumental variable (IV) methodology to try to obtain the causal effect of BMI on the set of health and school outcomes. The IV approach has been used by many authors to solve the endogeneity problem of BMI (for instance [37–39]).

We attempt to address the potential endogeneity of BMI by using the frequency of alcohol consumption as instrumental variable (${Z}_{i}$). In particular, the question that students’ answer in the HBSC survey is: “On how many days (if any) have you drink alcohol in the last 30 days?”, with seven possible answers (never, 1–2 days, 3–5 days, 6–9 days, 10–19 days, 20–29 days and 30 days or more). This categorical variable has been recoded as a set of binary variables.

To ensure the proper application of this methodology, the instrument must satisfy a number of conditions. In Appendix B, we detail the relevant properties as well as their application to this context.

Due to the categorical nature of the outcomes, we do not directly apply the two-stage least squares (2SLS) estimation to fit the data. Instead, the model is estimated using a conditional (recursive) mixed process estimator. The base model is composed of two equations:

2 $${O}_{i}^{*}=\alpha +\beta {BMI}_{i}+\gamma {X}_{i}+\delta {S}_{i}+{\varepsilon }_{i}$$

3 $${BMI}_{i}=\theta +\vartheta {Z}_{i}+\pi {X}_{i}+\rho {S}_{i}+{\omega }_{i}$$

where ${Z}_{i}$ is the frequency of alcohol consumption, ${\varepsilon }_{i}$ and ${\omega }_{i}$ are idiosyncratic random error terms for each equation. Equation (3) would be some kind of first stage in 2SLS, while Eq. (2) would be reduced form. The dependent variable in Eq. (2) is an ordered outcome and it is estimated using ordered probit model. The dependent variable in Eq. (3) is continuous and the model is estimated using Ordinary Least Squares (OLS). The inference using this structural equation model exploits the presence of an instrumental variable through its inclusion only in Eq. (3), which is correlated with the outcome only through its effect on the BMI. This is implemented using the Stata command *cmp* [40], which uses a canned routine to assure that standard errors are correctly estimated.

## Results

First, we estimate the influence of BMI on health outcomes, bullying and peer support assuming that BMI is exogenous—Eq. (1) in the methodology section. Second, we implement an instrumental variables (IV) estimator that addresses the concern about the endogeneity of BMI—Eqs. (2 and 3) in the methodology section. The full set of estimates is presented in the Appendix (Tables A4, A5 and A6). Given that probit coefficients have not a meaningful interpretation far from the sign, we have computed average marginal effects to know how large and important differences are. Tables 1, 2, 3, 4, 5 and 6 show average marginal effects for the key variable (BMI). Each category of the dependent variables requires a separate estimation, which will be denoted by k. For instance, “headache” will present $k=0, 1, 2, 3, 4$ marginal effects estimations. Tables also report the standard errors, which can be used to calculate the confidence intervals at a specific significance level:

4 $${CI}_{\beta ,\alpha }=[\widehat{\beta }\pm {z}_{1-\frac{\alpha }{2}}\bullet SE\left(\widehat{\beta }\right)]$$

where $\widehat{\beta }$ is the estimated average coefficient;$\alpha$ is the significance level; $SE$ is the standard error; and ${z}_{1-\frac{\alpha }{2}}$ is the percentile of the normal distribution.

**Table 1:** The effect of BMI on health and life satisfaction outcomes: Assuming that BMI is exogenous

Equation (1). Health and life satisfaction outcomes									
		Headache	Stomachache	Backache	Nervous	Difficulties in sleeping	Feeling dizzy	General health	Life satisfaction
BMI coefficient	*k* = 0	− 0.011***	− 0.003**	− 0.013***	− 0.004***	− 0.006***	− 0.005***	0.001***	0.001***
		(0.001)	(0.001)	(0.002)	(0.001)	(0.001)	(0.001)	(0.000)	(0.000)
		*0.000*	*0.017*	*0.000*	*0.001*	*0.000*	*0.000*	*0.000*	*0.000*
	*k* = 1	0.002***	0.001**	0.003***	0.000**	0.001***	0.001***	0.007***	0.000***
		(0.000)	(0.001)	(0.000)	(0.000)	(0.000)	(0.000)	(0.001)	(0.000)
		*0.000*	*0.018*	*0.000*	*0.016*	*0.000*	*0.000*	*0.000*	*0.000*
	*k* = 2	0.002***	0.001**	0.002***	0.001***	0.001***	0.001***	0.016***	0.001***
		(0.000)	(0.000)	(0.000)	(0.000)	(0.000)	(0.000)	(0.001)	(0.000)
		*0.000*	*0.017*	*0.000*	*0.001*	*0.000*	*0.000*	*0.000*	*0.000*
	*k* = 3	0.004***	0.001**	0.003***	0.001***	0.001***	0.001***	− 0.025***	0.001***
		(0.001)	(0.000)	(0.000)	(0.000)	(0.000)	(0.000)	(0.001)	(0.000)
		*0.000*	*0.018*	*0.000*	*0.001*	*0.000*	*0.001*	*0.000*	*0.000*
	*k* = 4	0.003***	0.001**	0.005***	0.002***	0.002***	0.001***		0.001***
		(0.000)	(0.000)	(0.001)	(0.001)	(0.001)	(0.000)		(0.000)
		*0.000*	*0.018*	*0.000*	*0.001*	*0.000*	*0.001*		*0.000*
	*k* = 5								0.004***
									(0.000)
									*0.000*
	*k* = 6								0.003***
									(0.000)
									*0.000*
	*k* = 7								0.004***
									(0.000)
									*0.000*
	*k* = 8								0.001***
									(0.000)
									*0.000*
	*k* = 9								− 0.004***
									(0.000)
									*0.000*
	*k* = 10								− 0.012***
									(0.001)
									*0.000*
Controls		Yes	Yes	Yes	Yes	Yes	Yes	Yes	Yes
Observations		9,369	9,363	9,341	9,338	9,335	9,316	9,487	9,435

Dependent variable: ordinal dependent variables

Method of estimation: Maximum Likelihood. Ordered probit model

Coefficient referred to marginal effects by category of the outcome (*k* = 0 up to 10)

Standard errors are in parentheses and are clustered at school level

Models control for students’ sex, immigrant status, parental occupation, household possessions, students’ perceptions of family well-off, frequency of doing vigorous physical activity and the hours of exercise per week

Source: Authors’ own calculations

Coefficient ***significant at 1%, ** at 5%, * at 10%. *p*-values are in italic format

**Table 2:** The effect of BMI on health and life satisfaction outcomes: IV approach

Equation (2). Health and life satisfaction outcomes									
		Headache	Stomachache	Backache	Nervous	Difficulties in sleeping	Feeling dizzy	General health	Life satisfaction
BMI coefficient	*k* = 0	− 0.073***	− 0.067***	− 0.077***	− 0.071***	− 0.064***	− 0.068***	0.030***	0.035***
		(0.004)	(0.004)	(0.003)	(0.004)	(0.006)	(0.004)	(0.006)	(0.005)
		*0.000*	*0.000*	*0.000*	*0.000*	*0.000*	*0.000*	*0.000*	*0.000*
	*k* = 1	0.007***	0.013***	0.007***	0.002***	0.007***	0.009***	0.024***	0.003***
		(0.001)	(0.001)	(0.001)	(0.000)	(0.001)	(0.001)	(0.001)	(0.001)
		*0.000*	*0.000*	*0.000*	*0.000*	*0.000*	*0.000*	*0.000*	*0.000*
	*k* = 2	0.005***	0.006***	0.006***	0.006***	0.006***	0.007***	0.028***	0.003***
		(0.001)	(0.000)	(0.001)	(0.000)	(0.000)	(0.001)	(0.003)	(0.001)
		*0.000*	*0.000*	*0.000*	*0.000*	*0.000*	*0.000*	*0.000*	*0.000*
	*k* = 3	0.017***	0.015***	0.010***	0.011***	0.010***	0.012***	− 0.082***	0.004***
		(0.001)	(0.001)	(0.001)	(0.001)	(0.001)	(0.001)	(0.003)	(0.000)
		*0.000*	*0.000*	*0.000*	*0.000*	*0.000*	*0.000*	*0.000*	*0.000*
	*k* = 4	0.043***	0.034***	0.054***	0.051***	0.040***	0.040***		0.006***
		(0.006)	(0.006)	(0.005)	(0.005)	(0.007)	(0.006)		(0.001)
		*0.000*	*0.000*	*0.000*	*0.000*	*0.000*	*0.000*		*0.000*
	*k* = 5								0.011***
									(0.001)
									*0.000*
	*k* = 6								0.007***
									(0.001)
									*0.000*
	*k* = 7								0.008***
									(0.001)
									*0.000*
	*k* = 8								0.004***
									(0.000)
									*0.000*
	*k* = 9								-0.004***
									(0.001)
									*0.000*
	*k* = 10								− 0.077***
									(0.003)
									*0.000*
Controls		Yes	Yes	Yes	Yes	Yes	Yes	Yes	Yes

Equation (3). BMI equation									
Alcohol consumption (Ref: Never)									
1–2 days		0.963***	0.971***	0.998***	1.012***	0.901***	0.913***	0.950***	0.994***
		(0.107)	(0.106)	(0.105)	(0.107)	(0.119)	(0.110)	(0.107)	(0.105)
		*0.000*	*0.000*	*0.000*	*0.000*	*0.000*	*0.000*	*0.000*	*0.000*
3–5 days		1.167***	1.064***	1.112***	1.074***	1.129***	1.159***	1.002***	1.091***
		(0.143)	(0.155)	(0.133)	(0.146)	(0.153)	(0.143)	(0.158)	(0.132)
		*0.000*	*0.000*	*0.000*	*0.000*	*0.000*	*0.000*	*0.000*	*0.000*
6–9 days		1.114***	1.211***	1.093***	1.027***	1.120***	1.109***	1.063***	0.973***
		(0.194)	(0.181)	(0.176)	(0.189)	(0.194)	(0.177)	(0.180)	(0.158)
		*0.000*	*0.000*	*0.000*	*0.000*	*0.000*	*0.000*	*0.000*	*0.000*
10–19 days		1.677***	1.793***	1.514***	1.491***	1.875***	1.967***	1.887***	1.397***
		(0.416)	(0.398)	(0.372)	(0.409)	(0.428)	(0.386)	(0.372)	(0.340)
		*0.000*	*0.000*	*0.000*	*0.000*	*0.000*	*0.000*	*0.000*	*0.000*
20–29 days		0.834	1.357**	1.097	1.523**	1.218*	1.493**	1.833***	0.569
		(0.669)	(0.672)	(0.697)	(0.619)	(0.726)	(0.682)	(0.516)	(0.375)
		*0.213*	*0.043*	*0.116*	*0.014*	*0.094*	*0.029*	*0.000*	*0.129*
30 days (or more)		0.638**	0.484	0.613*	0.957***	1.102***	0.558*	0.806**	0.916***
		(0.320)	(0.371)	(0.343)	(0.372)	(0.379)	(0.311)	(0.359)	(0.336)
		*0.046*	*0.191*	*0.074*	*0.010*	*0.004*	*0.073*	*0.025*	*0.006*
Missing flag		0.320**	0.404***	0.512***	0.468***	0.467***	0.426***	0.622***	0.619***
		(0.147)	(0.148)	(0.131)	(0.146)	(0.155)	(0.146)	(0.134)	(0.123)
		*0.030*	*0.006*	*0.000*	*0.001*	*0.003*	*0.004*	*0.000*	*0.000*
Controls		Yes	Yes	Yes	Yes	Yes	Yes	Yes	Yes
Observations		9,369	9,363	9,341	9,338	9,335	9,316	9,487	9,435

Dependent variable: ordinal dependent variables in Eq. (2) and Body Mass Index (continuous variable) in Eq. (3)

Method of estimation: conditional (recursive) mixed process estimator. Ordered probit model employed in Eq. (2) and OLS in Eq. (3). In Eq. (2), coefficient referred to marginal effects by category of the outcome (*k* = 0 up to 10)

Standard errors are in parentheses and are clustered at school level

Models control for students’ sex, immigrant status, parental occupation, household possessions, students’ perceptions of family well-off, frequency of doing vigorous physical activity and the hours of exercise per week

Source: Authors’ own calculations

For full set of parameter estimates see Table A4 (Appendix)

Coefficient ***significant at 1%, ** at 5%, * at 10%. *p*-values are in italic format

**Table 3:** The effect of BMI on fighting and bullying outcomes: Assuming that BMI is exogenous

Equation (1). Fighting and bullying outcomes						
		Physical fight	Bullying others	Been bullied	Cyberbullied by messages	Cyberbullied by pictures
BMI coefficient	*k* = 0	− 0.001	− 0.003***	− 0.001	− 0.001	− 0.001
		(0.002)	(0.001)	(0.001)	(0.001)	(0.001)
		*0.545*	*0.004*	*0.544*	*0.381*	*0.146*
	*k* = 1	0.000	0.002***	0.000	0.000	0.001
		(0.001)	(0.001)	(0.001)	(0.001)	(0.000)
		*0.546*	*0.004*	*0.544*	*0.381*	*0.144*
	*k* = 2	0.000	0.001***	0.000	0.000	0.000
		(0.000)	(0.000)	(0.000)	(0.000)	(0.000)
		*0.546*	*0.005*	*0.544*	*0.381*	*0.149*
	*k* = 3	0.000	0.000***	0.000	0.000	0.000
		(0.000)	(0.000)	(0.000)	(0.000)	(0.000)
		*0.545*	*0.008*	*0.546*	*0.393*	*0.162*
	*k* = 4	0.000	0.000***	0.000	0.000	0.000
		(0.000)	(0.000)	(0.000)	(0.000)	(0.000)
		*0.545*	*0.006*	*0.546*	*0.376*	*0.149*
Controls		Yes	Yes	Yes	Yes	Yes
Observations		6,064	8,461	8,516	8,380	8,355

Dependent variable: ordinal dependent variables

Method of estimation: Maximum Likelihood. Ordered probit model

Coefficient referred to marginal effects by category of the outcome (*k* = 0, 1, 2, 3, 4)

Standard errors are in parentheses and are clustered at school level

Models control for students’ sex, immigrant status, parental occupation, household possessions, students’ perceptions of family well-off, frequency of doing vigorous physical activity and the hours of exercise per week

Source: Authors’ own calculations

Coefficient ***significant at 1%, ** at 5%, * at 10%. *p*-values are in italic format

**Table 4:** The effect of BMI on fighting and bullying outcomes: IV approach

Equation (2). Fighting and bullying outcomes						
		Physical fight	Bullying others	Been bullied	Cyberbullied by messages	Cyberbullied by pictures
BMI coefficient	*k* = 0	− 0.062***	− 0.058***	0.019*	− 0.032***	− 0.037***
		(0.007)	(0.005)	(0.011)	(0.010)	(0.008)
		*0.000*	*0.000*	*0.087*	*0.001*	*0.000*
	*k* = 1	0.007***	0.018***	− 0.010**	0.008***	0.009***
		(0.002)	(0.001)	(0.005)	(0.001)	(0.001)
		*0.000*	*0.000*	*0.044*	*0.000*	*0.000*
	*k* = 2	0.008***	0.009***	− 0.003*	0.006***	0.006***
		(0.001)	(0.001)	(0.002)	(0.001)	(0.001)
		*0.000*	*0.000*	*0.085*	*0.000*	*0.000*
	*k* = 3	0.006***	0.007***	− 0.002	0.004***	0.004***
		(0.001)	(0.001)	(0.001)	(0.001)	(0.001)
		*0.000*	*0.000*	*0.117*	*0.000*	*0.000*
	*k* = 4	0.042***	0.024***	− 0.004	0.014**	0.018***
		(0.008)	(0.006)	(0.003)	(0.007)	(0.007)
		*0.000*	*0.000*	*0.201*	*0.048*	*0.009*
Controls		Yes	Yes	Yes	Yes	Yes

Equation (3). BMI equation						
Alcohol consumption (Ref: Never)						
1–2 days		0.899***	0.911***	0.999***	0.931***	0.945***
		(0.122)	(0.113)	(0.105)	(0.117)	(0.110)
		*0.000*	*0.000*	*0.000*	*0.000*	*0.000*
3–5 days		1.082***	1.053***	1.088***	1.146***	1.135***
		(0.164)	(0.165)	(0.184)	(0.168)	(0.174)
		*0.000*	*0.000*	*0.000*	*0.000*	*0.000*
6–9 days		1.176***	1.078***	0.981***	0.910***	1.016***
		(0.218)	(0.213)	(0.232)	(0.222)	(0.209)
		*0.000*	*0.000*	*0.000*	*0.000*	*0.000*
10–19 days		1.855***	2.003***	1.820***	1.930***	1.916***
		(0.440)	(0.398)	(0.487)	(0.451)	(0.441)
		*0.000*	*0.000*	*0.000*	*0.000*	*0.000*
20–29 days		1.921***	1.889***	1.840*	1.808**	1.567*
		(0.732)	(0.591)	(1.029)	(0.858)	(0.910)
		*0.009*	*0.001*	*0.074*	*0.035*	*0.085*
30 days (or more)		0.882**	0.769**	0.475	0.797**	0.589*
		(0.390)	(0.364)	(0.377)	(0.377)	(0.353)
		*0.024*	*0.035*	*0.208*	*0.035*	*0.095*
Missing flag		0.545***	0.591***	0.416**	0.538***	0.509***
		(0.163)	(0.153)	(0.185)	(0.173)	(0.162)
		*0.001*	*0.000*	*0.025*	*0.002*	*0.002*
Controls		Yes	Yes	Yes	Yes	Yes
Observations		6,064	8,461	8,516	8,380	8,355

Dependent variable: ordinal dependent variables in Eq. (2) and Body Mass Index (continuous variable) in Eq. (3)

Method of estimation: conditional (recursive) mixed process estimator. Ordered probit model employed in Eq. (2) and OLS in Eq. (3). In Eq. (2), coefficient referred to marginal effects by category of the outcome (*k* = 0, 1, 2, 3, 4)

Standard errors are in parentheses and are clustered at school level

Models control for students’ sex, immigrant status, parental occupation, household possessions, students’ perceptions of family well-off, frequency of doing vigorous physical activity and the hours of exercise per week

Source: Authors’ own calculations

For full set of parameter estimates see Table A5 (Appendix)

Coefficient ***significant at 1%, ** at 5%, * at 10%. *p*-values are in italic format

**Table 5:** The effect of BMI on peers’ support. Marginal effects: Assuming that BMI is exogenous

Equation (1). Peers support outcomes					
		Friends try to help	Can count on friends	Friends to share joys with	Can talk about problems with friends
BMI coefficient	*k* = 1	0.002***	0.002***	0.002***	0.002***
		(0.000)	(0.000)	(0.000)	(0.000)
		*0.000*	*0.000*	*0.000*	*0.000*
	*k* = 2	0.001***	0.001***	0.001***	0.001***
		(0.000)	(0.000)	(0.000)	(0.000)
		*0.000*	*0.000*	*0.000*	*0.000*
	*k* = 3	0.002***	0.001***	0.001***	0.001***
		(0.000)	(0.000)	(0.000)	(0.000)
		*0.000*	*0.000*	*0.000*	*0.000*
	*k* = 4	0.002***	0.002***	0.001***	0.001***
		(0.000)	(0.000)	(0.000)	(0.000)
		*0.000*	*0.000*	*0.000*	*0.000*
	*k* = 5	0.002***	0.002***	0.001***	0.001***
		(0.000)	(0.000)	(0.000)	(0.000)
		*0.000*	*0.000*	*0.000*	*0.000*
	*k* = 6	0.001***	0.001***	0.001***	0.001***
		(0.000)	(0.000)	(0.000)	(0.000)
		*0.000*	*0.000*	*0.000*	*0.001*
	*k* = 7	− 0.010***	− 0.009***	− 0.008***	− 0.006***
		(0.001)	(0.001)	(0.001)	(0.002)
		*0.000*	*0.000*	*0.000*	*0.000*
Controls		Yes	Yes	Yes	Yes
Observations		8,693	8,676	8,659	8,626

Dependent variable: ordinal dependent variables

Method of estimation: Maximum Likelihood. Ordered probit model

Coefficient referred to marginal effects by category of the outcome (*k* = 1 very strongly disagree; *k* = 7 very strongly agree)

Standard errors are in parentheses and are clustered at school level

Models control for students’ sex, immigrant status, parental occupation, household possessions, students’ perceptions of family well-off, frequency of doing vigorous physical activity and the hours of exercise per week

Source: Authors’ own calculations

Coefficient ***significant at 1%, ** at 5%, * at 10%. *p*-values are in italic format

**Table 6:** The effect of BMI on peers’ support. Marginal effects: IV approach

Equation (2). Peers support outcomes					
		Friends try to help	Can count on friends	Friends to share joys with	Can talk about problems with friends
BMI coefficient	*k* = 1	0.011***	0.007**	− 0.001	− 0.004
		(0.004)	(0.003)	(0.003)	(0.004)
		*0.005*	*0.017*	*0.826*	*0.295*
	*k* = 2	0.005***	0.004***	− 0.000	− 0.002
		(0.001)	(0.001)	(0.001)	(0.001)
		*0.000*	*0.002*	*0.825*	*0.267*
	*k* = 3	0.006***	0.004***	− 0.000	− 0.002
		(0.001)	(0.001)	(0.001)	(0.001)
		*0.000*	*0.001*	*0.825*	*0.260*
	*k* = 4	0.008***	0.006***	− 0.000	− 0.002
		(0.002)	(0.002)	(0.002)	(0.002)
		*0.000*	*0.001*	*0.824*	*0.253*
	*k* = 5	0.007***	0.005***	− 0.000	− 0.002
		(0.001)	(0.001)	(0.002)	(0.002)
		*0.000*	*0.001*	*0.824*	*0.245*
	*k* = 6	0.002***	0.002***	− 0.000	− 0.001
		(0.001)	(0.001)	(0.002)	(0.001)
		*0.001*	*0.003*	*0.8254*	*0.236*
	*k* = 7	− 0.040***	− 0.029***	0.003	0.012
		(0.009)	(0.010)	(0.012)	(0.011)
		*0.000*	*0.003*	*0.825*	*0.266*
Controls		Yes	Yes	Yes	Yes

Equation (3). BMI equation					
Alcohol consumption (Ref: Never)		Friends try to help	Can count on friends	Friends to share joys with	Can talk about problems with friends
1–2 days		0.975***	0.978***	0.982***	0.987***
		(0.112)	(0.110)	(0.108)	(0.107)
		*0.000*	*0.000*	*0.000*	*0.000*
3–5 days		1.101***	1.108***	1.103***	1.084***
		(0.177)	(0.181)	(0.184)	(0.187)
		*0.000*	*0.000*	*0.000*	*0.000*
6–9 days		0.879***	0.926***	0.972***	0.981***
		(0.227)	(0.222)	(0.235)	(0.233)
		*0.000*	*0.000*	*0.000*	*0.000*
10–19 days		1.973***	1.931***	1.860***	1.859***
		(0.464)	(0.481)	(0.498)	(0.490)
		*0.000*	*0.000*	*0.000*	*0.000*
20–29 days		1.778*	1.830*	1.880*	1.921**
		(0.971)	(0.989)	(0.977)	(0.956)
		*0.067*	*0.064*	*0.054*	*0.044*
30 days (or more)		0.265	0.347	0.461	0.491
		(0.371)	(0.385)	(0.385)	(0.377)
		*0.476*	*0.368*	*0.232*	*0.192*
Missing flag		0.514***	0.469***	0.461**	0.459**
		(0.175)	(0.180)	(0.186)	(0.183)
		*0.003*	*0.009*	*0.013*	*0.012*
Controls		Yes	Yes	Yes	Yes
Observations		8,693	8,676	8,659	8,626

Dependent variable: ordinal dependent variables in Eq. (2) and Body Mass Index (continuous variable) in Eq. (3)

Method of estimation: conditional (recursive) mixed process estimator. Ordered probit model employed in Eq. (2) and OLS in Eq. (3). In Eq. (2), coefficient referred to marginal effects by category of the outcome (*k* = 1 very strongly disagree; *k* = 7 very strongly agree)

Standard errors are in parentheses and are clustered at school level

Models control for students’ sex, immigrant status, parental occupation, household possessions, students’ perceptions of family well-off, frequency of doing vigorous physical activity and the hours of exercise per week

Source: Authors’ own calculations

For full set of parameter estimates see Table A6 (Appendix)

Coefficient ***significant at 1%, ** at 5%, * at 10%. *p*-values are in italic format

Tables also incorporate *p*-values and significance levels denoted by asterisks to facilitate the interpretation of the results (*** for 1%, ** for 5%, and * for 10%).

### Health and life satisfaction

To begin, Tables 1 and 2 model the influence of BMI on health complaints, self-assessment of health and life satisfaction, controlling for socioeconomic and demographic factors. First, we assume that BMI is exogenous (Table 1). In this case, we identified that BMI is positively associated with health complaints but negatively associated with self-assessment of health and life satisfaction.

Then, we account for the endogeneity of BMI (Table 2). The direction of the association between BMI and the outcomes does not change, but coefficient estimates of BMI are considerably higher for IV than non-IV. For instance, one-point increase in the BMI raises the likelihood of having headaches more than once a week by 1.7% (average marginal effects for *k* = 3). The likelihood of reporting other psychosocial complaints more than once a week is very similar. With one-point increase of BMI, the likelihood of having backache increases by 1%, feeling nervous by 1.1%, having difficulties in sleeping by 1%, feeling dizzy by 1.2% and stomachache by 1.5%.

Regarding self-assessment of general health, IV specification points towards a significant and negative influence of BMI. In particular, students are 8.2 points less likely to say that their health is excellent with one-unit increase of BMI. Similarly, BMI is negatively associated with students’ subjective well-being. One-point increase of BMI decreases the likelihood of having “best possible life” by 7.7%.

Lastly, Eq. (3) models students’ BMI conditional on alcohol consumption and socioeconomic and demographic variables. As observed, the frequency of alcohol consumption is significantly associated with BMI. Just having drunk alcohol during the last 30 days once or twice—which could be considered a small frequency compared to the reference category (not having drunk)—seems to be positively associated with BMI. Particularly, it increases BMI by 1 point. This result holds for all the specifications.

### Fighting and bullying

Tables 3 and 4 show the influence of BMI on fighting and bullying indicators, adjusted for socioeconomic and demographic characteristics. In Table 3, the association between BMI and the variables under investigation is explored using ordered probit estimations. According to these models, it seems that BMI does not influence students’ participation in fights nor does the likelihood of suffering bullying. We only found a significant positive association (although weak) of BMI in the probability of bullying other peers.

However, when instrumenting the BMI (Table 4), its influence turns significant. As shown in Eq. (2) (IV approach), one-point increase of BMI increases the likelihood of participating in fights 4 times or more during the last 12 months by 4.2% (average marginal effects for *k* = 4). In the same way, having a higher BMI is positively associated with perpetrating bullying against others. The average marginal effect of BMI is equal to 2.4% increase in the likelihood of bullying other peers more than once a week (*k* = 4).

Regarding experiences of being bullied, results show that an increase of BMI leads to lower probability of being a victim of bullying. In particular, the increase of BMI is associated with 1.9% likelihood of not being a victim of bullying (*k* = 0). Conversely, when it comes to cyberbullying, BMI is positively associated. Specifically, one-point increase of BMI is associated with 1.4% higher likelihood of being cyberbullied by messages several times a week (e.g., instant messages, wall postings, emails and text messages, or created a website) (*k* = 4) and 1.8% of being photographed without permission and the pictures posted online.

It is worth noting that the general bullying question seems related to bullying happening in school, considering the definition provided in the HBSC questionnaire.^4^ Thus, higher BMI prevents students to be bullied in person (at school), but increase the likelihood of being bullied online. To the same extent, the greater physical build associated with higher BMI increases the probability of bullying other peers at school and being involved in physical fights.

### Social context: peers’ support

Tables 5 and 6 report the influence of BMI on peers’ support, using as indicators: friends’ help, trust in friends, share joys and sorrows with friends and can talk with friends about problems. In the first equation (BMI exogenous, Table 5), it seems that BMI is significantly and positively associated with all the measures of peers’ support.

However, considering BMI as exogenous may overestimate the influence of BMI on peers’ support. When modelling BMI, the association between BMI and the questions related with having friends to share joys and sorrows and talk about problems disappears (Table 6). Nevertheless, the association of BMI with the statements related to friends’ help and trust in friends remains negative and significant. In particular, results show that the likelihood of strongly agreeing (*k* = 7) with receiving friends’ help drops by 4% as a result of one-point increase of BMI. Similarly, it is also less likely that students strongly agree with the statement related to counting on friends when increasing BMI (average marginal effect of -2.9%).

## Discussion and conclusions

In this research, we have explored the association of BMI with perceived health, bullying and social support at school. In order to undertake this study, we have employed the Spanish records from the 2013–14 Health Behaviour in School-Aged Children Survey*.* With the aim of mitigating the endogeneity of BMI, we have employed an IV strategy, using alcohol consumption as instrumental variable. Other important strengths of the study are the large sample size and the array of socioeconomic and demographic controls.

The empirical evidence confirms that BMI conditions the daily life of Spanish students. Firstly, we found that BMI is associated with more psychosomatic complaints, such as backaches, headaches and sleeping disorders. This positive association between BMI and psychosomatic complaints had been described in other countries like Finland [41], Germany [42] or Sweden [43]. In addition to this, an increase of BMI is associated with lower life satisfaction; this effect is within the trend observed in western countries, in which excess of weight is regarded negatively, and hence reduces subjective well-being [44].

Secondly, BMI increases the frequency of participating in physical fights and bullying other students at school. Conversely to the previous studies for Spain [31, 32], we have found that BMI is not significantly associated with the risk of bullying victimization at school (face-to-face bullying). However, our results note that BMI is significantly associated with the bullying developed in the online realm (cyberbullying). This entails that overweight students suffer more verbal than physical abuse [45]. According to Kowalski et al. [46], the perceived anonymity of the aggressor and the higher accessibility are adverse effects of online bullying compared to face-to-face bullying, which justify its expansion.

The specific organization of a school day in Spain further illuminates the notable association of bullying victimization online and its absence during regular school hours. In Spain, the majority of schools follow a condensed schedule, with only morning sessions [47]. Consequently, the opportunities for in-person interactions out of the classroom are limited to the school break which is short, while they persist or even increase out-of-school, given the time that children spend connected to the Internet. In fact, Spanish adolescents spend most of their free time using the mobile phone, tablet or computers [48]. On average, children between 9 and 16 years old connect to the Internet more than 3 h a day in Spain, mainly for activities related to communication and entertainment [49].

Lastly, results show that the increase of BMI makes more difficult to feel peer support. Specifically, a weight gain reduces the chances of feeling friend’s help and counting on friends. The social isolation experienced by overweight students might be based on the *weight stigma*, which acts as a foundation for social disapproval: “overweight or obesity are negatively stereotyped as being lazy, lacking willpower and self-discipline, unmotivated to improve their health” (p. 402, [50]. An underlying driver of the lower social support might be the constant exposure to unrealistic beauty standards prevalent on the Internet. In particular, Calado et al. [51] highlight a significant association between exposure kinds of media topics related to body image—such as dieting, fashion, and fitness—and body dissatisfaction, which affects more to female than male.

Policy interventions resulting from these findings may be driven in different ways. On the one hand, they should be focused on helping children to achieve a healthy BMI. As it happens in other countries, such as the United States, health assessment at school can be a useful tool to inform parents and children about healthy weight ranges. In Spain, given that medical check-ups are available through the National Health System, there is no health assessment at school. However, we find it relevant to develop this action at school, since it ensures to reach the target group.

On the other hand, the high prevalence of overweight among Spanish youth may be attributed to their lifestyle. Research by Grosso and Galvano [52] demonstrated a low adherence to the Mediterranean diet among children and adolescents in Spain and a decreasing trend in this adherence. This relationship was mediated by various social and demographic factors; notably, individuals from socioeconomically advantaged backgrounds tended to exhibit higher adherence to the diet. In this context, a second recommendation is to implement nutrition education programs at school to promote healthy eating habits from a young age, paying special attention to low-income schools.

In addition, this study alerts to higher discrimination as a result of the increase of BMI, particularly in online environments. Consequently, policy actions should be targeted at dealing with the social stigma of overweight. Cultivating a culture of non-discrimination within school is a challenge that involves the entire educational community. In the classroom, there are various approaches, like discussing and identifying weight stereotypes, designing mechanisms to monitor incidents of discrimination or involving students in peer mediation activities. Regarding the role of parents, they should pay particular attention to the time that children spend on the Internet using social networks which, together with an inadequate use, may foster cyberbullying. In this sense, enhancing parents’ awareness of children’s use of social networks can help prevent cyberbullying. A practical approach to monitoring online activity is to use parental control apps.

Some limitations apply to the present study. First of all, the use of IV tries to deal with the endogeneity problem of BMI, but there might be other unobservable variables that we cannot control for. For instance, prior research indicates a significant correlation between adolescent life satisfaction and family structure [53], a relationship that we currently lack information on. Secondly, it is worth noting that all measures were self-reported. While this approach is advisable for some variables, for others the results should be interpreted with caution. For example, when it comes to assessing bullying comprehensively, anonymous self-reports tend to be the most reliable, as school personnel and classmates may not always be aware of every instance [54]. However, when it comes to self-reporting weight and height, there can be a risk of misclassification. Previous investigations indicate a pattern of underreporting for weight, along with over-reporting for height, which leads to a lower BMI and tends to underestimate the prevalence of overweight and obesity [55].

Further research that incorporates objective measurements of height and weight is needed to investigate the impact of BMI on experiences of bullying and peer support in a more accurate way. Additionally, it is important to note that the majority of studies examining the relationship between BMI and the school context are correlational. While this research takes a step towards establishing causality, more causal statements are needed. To better address these questions, the implementation of longitudinal assessments throughout the school period would be particularly valuable.

### Supplementary Information

Below is the link to the electronic supplementary material.

Supplementary file1 (DOCX 103 kb)

Supplementary file2 (DOCX 17 kb)

## Data Availability

The database used in this research is available through the next link: https://hbsc.org/data/.

## Footnotes

1 Data retrieved from Global Obesity Observatory. Access link: https://data.worldobesity.org/tables/prevalence-of-child-overweight-including-obesity-3/

2 Drosopoulou et al. [33] conducted a correlational study which analysed the relationship between psychosocial health and body mass index including a sample of 1,492 Spanish adolescences. The sample size was quite smaller than the one used in this study (n = 11,136) and they did not try to approach a causal effect.

3 WHO (1946). Constitution of the World Health Organization. https://www.who.int/about/governance/ constitution.

4 The introduction to bullying questions in the HBSC questionnaire (2013-14) indicates: “We say a student is being bullied when another student, or a group of students, say or do nasty and unpleasant things to him or her. It is also bullying when a student is teased repeatedly in a way he or she does not like or when he or she is deliberately left out of things. But it is not bullying when two students of about the same strength or power argue or fight. It is also not bullying when a student is teased in a friendly and playful way”.
