# Supplementary material for: Influence of body mass index on health complains and life satisfaction
Source: Qual Life Res. 2023 Dec 1;33(3):705–19. doi: 10.1007/s11136-023-03557-0 (PMC10894113; doi:10.1007/s11136-023-03557-0)
Supplement: Supplementary file 1 — Supplementary file1 (DOCX 103 kb) [file 11136_2023_3557_MOESM1_ESM.docx]

**Appendix**

Table A1. Descriptive statistics. Sample used and sample not included.

|  |  | Sample used | | | Sample not included | | |
| --- | --- | --- | --- | --- | --- | --- | --- |
|  |  | Mean | S.D. | Obs. | Mean | S.D. | Obs. |
| Body Mass Index (BMI) |  | 19.800 | 3.276 | 9,565 | - | - | - |
| In the last 6 months: how often have you had headache? | Rarely or never | 0.616 | 0.486 | 9,369 | 0.585 | 0.493 | 644 |
|  | About every month | 0.148 | 0.355 | 9,369 | 0.144 | 0.352 | 644 |
|  | About every week | 0.069 | 0.254 | 9,369 | 0.061 | 0.239 | 644 |
|  | More than once/week | 0.116 | 0.320 | 9,369 | 0.137 | 0.344 | 644 |
|  | About every day | 0.050 | 0.219 | 9,369 | 0.073 | 0.260 | 644 |
| In the last 6 months: how often have you had stomachache? | Rarely or never | 0.642** | 0.479 | 9,363 | 0.602** | 0.490 | 643 |
|  | About every month | 0.203* | 0.402 | 9,363 | 0.174* | 0.380 | 643 |
|  | About every week | 0.050 | 0.218 | 9,363 | 0.062 | 0.242 | 643 |
|  | More than once/week | 0.071*** | 0.258 | 9,363 | 0.109*** | 0.312 | 643 |
|  | About every day | 0.033*** | 0.179 | 9,363 | 0.053*** | 0.224 | 643 |
| In the last 6 months: how often have you had backache? | Rarely or never | 0.629 | 0.483 | 9,341 | 0.639 | 0.481 | 638 |
|  | About every month | 0.151* | 0.358 | 9,341 | 0.124* | 0.330 | 638 |
|  | About every week | 0.073 | 0.259 | 9,341 | 0.066 | 0.248 | 638 |
|  | More than once/week | 0.076 | 0.265 | 9,341 | 0.080 | 0.271 | 638 |
|  | About every day | 0.072* | 0.258 | 9,341 | 0.091* | 0.288 | 638 |
| In the last 6 months: how often have you felt nervous? | Rarely or never | 0.414 | 0.493 | 9,338 | 0.439 | 0.497 | 635 |
|  | About every month | 0.216 | 0.412 | 9,338 | 0.191 | 0.393 | 635 |
|  | About every week | 0.127 | 0.333 | 9,338 | 0.128 | 0.334 | 635 |
|  | More than once/week | 0.126* | 0.331 | 9,338 | 0.101* | 0.301 | 635 |
|  | About every day | 0.117* | 0.321 | 9,338 | 0.142* | 0.349 | 635 |
| In the last 6 months: how often have you had difficulties in  getting to sleep? | Rarely or never | 0.683 | 0.465 | 9,335 | 0.658 | 0.475 | 637 |
|  | About every month | 0.117 | 0.321 | 9,335 | 0.108 | 0.311 | 637 |
|  | About every week | 0.061 | 0.240 | 9,335 | 0.057 | 0.231 | 637 |
|  | More than once/week | 0.067 | 0.250 | 9,335 | 0.077 | 0.267 | 637 |
|  | About every day | 0.071*** | 0.257 | 9,335 | 0.100*** | 0.301 | 637 |
| In the last 6 months: how often have you felt dizzy? | Rarely or never | 0.714 | 0.452 | 9,316 | 0.728 | 0.445 | 633 |
|  | About every month | 0.128* | 0.334 | 9,316 | 0.103* | 0.304 | 633 |
|  | About every week | 0.055 | 0.229 | 9,316 | 0.047 | 0.213 | 633 |
|  | More than once/week | 0.060 | 0.238 | 9,316 | 0.054 | 0.226 | 633 |
|  | About every day | 0.042*** | 0.201 | 9,316 | 0.068*** | 0.252 | 633 |
| Would you say your health is…? | Poor | 0.008* | 0.088 | 9,487 | 0.015* | 0.120 | 619 |
|  | Fair | 0.066*** | 0.248 | 9,487 | 0.094*** | 0.292 | 619 |
|  | Good | 0.498 | 0.500 | 9,487 | 0.502 | 0.500 | 619 |
|  | Excellent | 0.428* | 0.495 | 9,487 | 0.389* | 0.488 | 619 |
| Life satisfaction.  Here is a picture of a ladder. The top of the ladder '10' is the best possible life for you and the bottom '0' is the worst possible life for you. In general, where on the ladder do you feel you stand at the moment? Tick the box next to the number that best describes where you stand. | 0, Worst possible life | 0.009*** | 0.096 | 9,435 | 0.028*** | 0.165 | 607 |
|  | 1 | 0.005 | 0.070 | 9,435 | 0.005 | 0.070 | 607 |
|  | 2 | 0.007* | 0.081 | 9,435 | 0.013* | 0.114 | 607 |
|  | 3 | 0.012** | 0.107 | 9,435 | 0.023** | 0.150 | 607 |
|  | 4 | 0.022 | 0.146 | 9,435 | 0.020 | 0.139 | 607 |
|  | 5 | 0.068** | 0.251 | 9,435 | 0.089** | 0.285 | 607 |
|  | 6 | 0.071 | 0.257 | 9,435 | 0.072 | 0.260 | 607 |
|  | 7 | 0.148 | 0.355 | 9,435 | 0.133 | 0.340 | 607 |
|  | 8 | 0.230*** | 0.421 | 9,435 | 0.183*** | 0.387 | 607 |
|  | 9 | 0.204*** | 0.403 | 9,435 | 0.155*** | 0.362 | 607 |
|  | 10, Best possible life | 0.224*** | 0.417 | 9,435 | 0.278*** | 0.449 | 607 |
| During the past 12 months, how many times were you in a physical fight? | None | 0.701*** | 0.458 | 6,064 | 0.642*** | 0.480 | 452 |
|  | 1 time | 0.154 | 0.361 | 6,064 | 0.170 | 0.376 | 452 |
|  | 2 times | 0.069 | 0.254 | 6,064 | 0.060 | 0.237 | 452 |
|  | 3 times | 0.031*** | 0.175 | 6,064 | 0.055*** | 0.229 | 452 |
|  | 4 times or more | 0.045*** | 0.207 | 6,064 | 0.073*** | 0.260 | 452 |
| How often have you taken part in bullying another student(s) at school in the past couple of months? | Haven't | 0.828* | 0.377 | 8,461 | 0.800* | 0.401 | 639 |
|  | Once or twice | 0.123 | 0.328 | 8,461 | 0.127 | 0.333 | 639 |
|  | 2-3 times per month | 0.026 | 0.161 | 8,461 | 0.034 | 0.182 | 639 |
|  | Once a week | 0.012 | 0.108 | 8,461 | 0.013 | 0.111 | 639 |
|  | Several times a week | 0.011*** | 0.104 | 8,461 | 0.027*** | 0.161 | 639 |
| How often have you been bullied at school in the past couple of months? | Haven't | 0.852*** | 0.356 | 8,516 | 0.777*** | 0.417 | 645 |
|  | Once or twice | 0.099** | 0.298 | 8,516 | 0.130** | 0.337 | 645 |
|  | 2-3 times per month | 0.023 | 0.151 | 8,516 | 0.033 | 0.178 | 645 |
|  | Once a week | 0.011*** | 0.106 | 8,516 | 0.029*** | 0.169 | 645 |
|  | Several times week | 0.015*** | 0.122 | 8,516 | 0.031*** | 0.173 | 645 |
| How often have you been bullied in the following ways?: Someone sent mean instant messages, wall postings, emails and text messages, or created a website that made fun of me. | Haven't | 0.926** | 0.263 | 8,380 | 0.901** | 0.299 | 617 |
|  | Once or twice | 0.036 | 0.186 | 8,380 | 0.039 | 0.194 | 617 |
|  | 2-3 times per month | 0.018 | 0.132 | 8,380 | 0.023 | 0.149 | 617 |
|  | Once week | 0.009** | 0.097 | 8,380 | 0.018** | 0.132 | 617 |
|  | Several times week | 0.012* | 0.107 | 8,380 | 0.019* | 0.138 | 617 |
| How often have you been bullied in the following ways? Someone took unflattering or inappropriate pictures of me without permission and posted them online. | Haven't | 0.926** | 0.262 | 8,355 | 0.902** | 0.297 | 624 |
|  | Once or twice | 0.037 | 0.190 | 8,355 | 0.040 | 0.196 | 624 |
|  | 2-3 times per month | 0.017* | 0.130 | 8,355 | 0.027* | 0.163 | 624 |
|  | Once a week | 0.008 | 0.089 | 8,355 | 0.010 | 0.098 | 624 |
|  | Several times week | 0.011** | 0.106 | 8,355 | 0.021** | 0.143 | 624 |
| My friends really try to help me | 1 Strongly disagree | 0.040*** | 0.196 | 8,693 | 0.062*** | 0.242 | 658 |
|  | 2 | 0.032 | 0.175 | 8,693 | 0.032 | 0.176 | 658 |
|  | 3 | 0.051* | 0.221 | 8,693 | 0.068* | 0.253 | 658 |
|  | 4 | 0.098* | 0.298 | 8,693 | 0.120* | 0.325 | 658 |
|  | 5 | 0.137** | 0.344 | 8,693 | 0.108** | 0.310 | 658 |
|  | 6 | 0.211*** | 0.408 | 8,693 | 0.157*** | 0.364 | 658 |
|  | 7 Strongly agree | 0.431 | 0.495 | 8,693 | 0.453 | 0.498 | 658 |
| I can count on my friends when things go wrong | 1 Strongly disagree | 0.042*** | 0.201 | 8,676 | 0.064*** | 0.246 | 653 |
|  | 2 | 0.036* | 0.185 | 8,676 | 0.049* | 0.216 | 653 |
|  | 3 | 0.047 | 0.212 | 8,676 | 0.052 | 0.222 | 653 |
|  | 4 | 0.088 | 0.283 | 8,676 | 0.104 | 0.306 | 653 |
|  | 5 | 0.118 | 0.323 | 8,676 | 0.107 | 0.310 | 653 |
|  | 6 | 0.212*** | 0.409 | 8,676 | 0.155*** | 0.362 | 653 |
|  | 7 Strongly agree | 0.457 | 0.498 | 8,676 | 0.469 | 0.499 | 653 |
| I have friends with whom I can share my joys and sorrows | 1 Strongly disagree | 0.052** | 0.223 | 8,659 | 0.072** | 0.259 | 652 |
|  | 2 | 0.032* | 0.176 | 8,659 | 0.044* | 0.206 | 652 |
|  | 3 | 0.036 | 0.187 | 8,659 | 0.043 | 0.203 | 652 |
|  | 4 | 0.072 | 0.259 | 8,659 | 0.080 | 0.271 | 652 |
|  | 5 | 0.095 | 0.294 | 8,659 | 0.080 | 0.271 | 652 |
|  | 6 | 0.175 | 0.380 | 8,659 | 0.163 | 0.369 | 652 |
|  | 7 Strongly agree | 0.537 | 0.499 | 8,659 | 0.518 | 0.500 | 652 |
| I can talk about my problems with my friends | 1 Strongly disagree | 0.058*** | 0.234 | 8,626 | 0.099*** | 0.299 | 648 |
|  | 2 | 0.035 | 0.184 | 8,626 | 0.039 | 0.193 | 648 |
|  | 3 | 0.044 | 0.204 | 8,626 | 0.056 | 0.229 | 648 |
|  | 4 | 0.081 | 0.273 | 8,626 | 0.085 | 0.279 | 648 |
|  | 5 | 0.102 | 0.303 | 8,626 | 0.096 | 0.294 | 648 |
|  | 6 | 0.187** | 0.390 | 8,626 | 0.154** | 0.362 | 648 |
|  | 7 Strongly agree | 0.493 | 0.500 | 8,626 | 0.472 | 0.500 | 648 |
| Sex | Female | 0.512** | 0.500 | 9,565 | 0.488** | 0.500 | 1,571 |
|  | Male | 0.488** | 0.500 | 9,565 | 0.512** | 0.500 | 1,571 |
| Father occupation | Low | 0.130** | 0.337 | 9,565 | 0.109** | 0.312 | 1,571 |
|  | Medium-Low | 0.286 | 0.452 | 9,565 | 0.295 | 0.456 | 1,571 |
|  | Medium | 0.045 | 0.207 | 9,565 | 0.040 | 0.196 | 1,571 |
|  | Medium-High | 0.075* | 0.263 | 9,565 | 0.061* | 0.240 | 1,571 |
|  | High | 0.142** | 0.349 | 9,565 | 0.122** | 0.327 | 1,571 |
|  | Unclassifiable | 0.322*** | 0.467 | 9,565 | 0.373*** | 0.484 | 1,571 |
| Mother occupation | Low | 0.110 | 0.313 | 9,565 | 0.115 | 0.319 | 1,571 |
|  | Medium-Low | 0.159*** | 0.365 | 9,565 | 0.132*** | 0.339 | 1,571 |
|  | Medium | 0.155*** | 0.362 | 9,565 | 0.115*** | 0.319 | 1,571 |
|  | Medium-High | 0.052* | 0.222 | 9,565 | 0.041* | 0.199 | 1,571 |
|  | High | 0.143 | 0.350 | 9,565 | 0.100 | 0.300 | 1,571 |
|  | Unclassifiable | 0.381*** | 0.486 | 9,565 | 0.496*** | 0.500 | 1,571 |
| Does your family own a car, van or truck? | None | 0.026** | 0.159 | 9,565 | 0.035** | 0.184 | 1,571 |
|  | One | 0.251*** | 0.434 | 9,565 | 0.167*** | 0.373 | 1,571 |
|  | Two or more | 0.401*** | 0.490 | 9,565 | 0.228*** | 0.420 | 1,571 |
|  | Missing flag | 0.323*** | 0.468 | 9,565 | 0.570*** | 0.495 | 1,571 |
| Do you have your own bedroom for yourself? | No | 0.118*** | 0.323 | 9,565 | 0.077*** | 0.267 | 1,571 |
|  | Yes | 0.557*** | 0.497 | 9,565 | 0.350*** | 0.477 | 1,571 |
|  | Missing flag | 0.325*** | 0.468 | 9,565 | 0.573*** | 0.495 | 1,571 |
| How many computers do your family own (including laptops and tablets, not including game consoles and smartphones)? | None | 0.011 | 0.102 | 9,565 | 0.010 | 0.097 | 1,571 |
|  | One | 0.091*** | 0.287 | 9,565 | 0.070*** | 0.255 | 1,571 |
|  | Two | 0.180*** | 0.384 | 9,565 | 0.106*** | 0.308 | 1,571 |
|  | More than two | 0.392*** | 0.488 | 9,565 | 0.241*** | 0.428 | 1,571 |
|  | Missing flag | 0.327*** | 0.469 | 9,565 | 0.573*** | 0.495 | 1,571 |
| How many bathrooms (room with a bath/shower or both) are in your home? | None | 0.003 | 0.058 | 9,565 | 0.005 | 0.071 | 1,571 |
|  | One | 0.193*** | 0.395 | 9,565 | 0.134*** | 0.340 | 1,571 |
|  | Two | 0.361*** | 0.480 | 9,565 | 0.209*** | 0.407 | 1,571 |
|  | More than two | 0.119*** | 0.324 | 9,565 | 0.080*** | 0.272 | 1,571 |
|  | Missing flag | 0.324*** | 0.468 | 9,565 | 0.572*** | 0.495 | 1,571 |
| Does your family have a dishwasher at home? | No | 0.190*** | 0.392 | 9,565 | 0.122*** | 0.327 | 1,571 |
|  | Yes | 0.487*** | 0.500 | 9,565 | 0.305*** | 0.461 | 1,571 |
|  | Missing flag | 0.324*** | 0.468 | 9,565 | 0.574*** | 0.495 | 1,571 |
| How many times did you and your family travel out of Spain for a holiday/vacation last year? | Not at all | 0.344*** | 0.475 | 9,565 | 0.213*** | 0.409 | 1,571 |
|  | Once | 0.194*** | 0.396 | 9,565 | 0.112*** | 0.316 | 1,571 |
|  | Twice | 0.071*** | 0.256 | 9,565 | 0.043*** | 0.202 | 1,571 |
|  | More than twice | 0.068 | 0.253 | 9,565 | 0.058 | 0.234 | 1,571 |
|  | Missing flag | 0.323*** | 0.467 | 9,565 | 0.575*** | 0.495 | 1,571 |
| How well-off do you think your family is? | not at all well-off | 0.009** | 0.093 | 9,565 | 0.004** | 0.062 | 1,571 |
|  | not very well-off | 0.052** | 0.222 | 9,565 | 0.039** | 0.195 | 1,571 |
|  | average | 0.827*** | 0.378 | 9,565 | 0.456*** | 0.498 | 1,571 |
|  | quite well-off | 0.075*** | 0.263 | 9,565 | 0.039*** | 0.195 | 1,571 |
|  | very well-off | 0.023 | 0.149 | 9,565 | 0.022 | 0.148 | 1,571 |
|  | Missing flag | 0.015*** | 0.121 | 9,565 | 0.439*** | 0.496 | 1,571 |
| Immigrant status | Native | 0.795*** | 0.404 | 9,565 | 0.750*** | 0.433 | 1,571 |
|  | Immigrant first | 0.098 | 0.298 | 9,565 | 0.111 | 0.315 | 1,571 |
|  | Immigrant second | 0.107*** | 0.309 | 9,565 | 0.139*** | 0.346 | 1,571 |
| Frequency of doing vigorous physical activity | Never | 0.061** | 0.239 | 9,565 | 0.046** | 0.211 | 1,571 |
|  | Less than once a month | 0.046*** | 0.210 | 9,565 | 0.031*** | 0.172 | 1,571 |
|  | Once a month | 0.038*** | 0.192 | 9,565 | 0.018*** | 0.135 | 1,571 |
|  | Once a week | 0.101*** | 0.301 | 9,565 | 0.044*** | 0.205 | 1,571 |
|  | 2-3 times a week | 0.303*** | 0.460 | 9,565 | 0.142*** | 0.349 | 1,571 |
|  | 4-6 times a week | 0.225*** | 0.417 | 9,565 | 0.073*** | 0.260 | 1,571 |
|  | Every day | 0.147*** | 0.354 | 9,565 | 0.064*** | 0.245 | 1,571 |
|  | Missing flag | 0.079*** | 0.270 | 9,565 | 0.582*** | 0.493 | 1,571 |
| Exercise - hours a week | None | 0.117*** | 0.321 | 9,565 | 0.071*** | 0.257 | 1,571 |
|  | Half an hour | 0.099*** | 0.298 | 9,565 | 0.060*** | 0.238 | 1,571 |
|  | 1 hour | 0.212*** | 0.409 | 9,565 | 0.100*** | 0.300 | 1,571 |
|  | 2-3 hours | 0.274*** | 0.446 | 9,565 | 0.109*** | 0.312 | 1,571 |
|  | 4-6 hours | 0.137*** | 0.344 | 9,565 | 0.039*** | 0.193 | 1,571 |
|  | 7 hours or more | 0.083*** | 0.276 | 9,565 | 0.034*** | 0.181 | 1,571 |
|  | Missing flag | 0.079*** | 0.269 | 9,565 | 0.587*** | 0.493 | 1,571 |
| Alcohol use last 30 days | Never | 0.796 | 0.403 | 9,565 | 0.798 | 0.402 | 1,571 |
|  | 1-2 days | 0.095* | 0.293 | 9,565 | 0.080* | 0.272 | 1,571 |
|  | 3-5 days | 0.038** | 0.192 | 9,565 | 0.027** | 0.163 | 1,571 |
|  | 6-9 days | 0.017 | 0.131 | 9,565 | 0.014 | 0.118 | 1,571 |
|  | 10-19 days | 0.007 | 0.085 | 9,565 | 0.009 | 0.094 | 1,571 |
|  | 20-29 days | 0.002 | 0.041 | 9,565 | 0.002 | 0.044 | 1,571 |
|  | 30 days (or more) | 0.005 | 0.069 | 9,565 | 0.008 | 0.087 | 1,571 |
|  | Missing flag | 0.040*** | 0.197 | 9,565 | 0.062*** | 0.242 | 1,571 |

Note: “Obs.” stands for “Observations” and “S.D.” stands for “Standard Deviations”. Authors’ own calculation based on the records of HBSC study 2013-14 for Spain. A test of mean differences in mean between the sample used and the sample not included has been performed. The null hypothesis is the equality of means between both samples. *** denotes significant differences to level 1%; ** to 5%; * to 10%. Source: Authors’ own calculation.

Table A2. Summary of the outcomes

| Subset | Particular outcome | Scale of the response |
| --- | --- | --- |
| **Health and life satisfaction** | Health psychosocial complaints: headache, stomachache, backache, feeling nervous, difficulties in sleeping and feeling dizzy. | Rarely or never, about every month, about every week, more than once/week and about every day, coded from 0 to 4, respectively. |
|  | Self-rated general health | “Excellent”, “good”, “fair” and “poor”, coded from 0 to 3. |
|  | Life satisfaction | Between 0 (worst possible life) and 10 (best possible life). |
| **Fighting and bullying** | Involvement in physical fights | “1 time”, “2 times”, “3 times” and “4 times or more”, coded from 0 to 4. |
|  | Bullying: the type of bullying they have experienced in that case (cyberbullied by messages and pictures), and also if they have perpetrated bullying | “Haven’t”, “once or twice”, “2-3 times per month”, “once a week” and “several times a week”, coded from 0 to 4. |
| **Peers’ support** | a) “My friends really try to help me”, b) “I can count on my friends when things go wrong”, c) “I have friends with whom I can share my joys and sorrows”, and d) “I can talk about my problems with my friends”. | Very strongly disagree (1) to very strongly agree (7). |

Source: Authors’ own elaboration based on HBSC study.

Table A3. Summary of demographic and additional control variables.

| Group | Subset | Variable | Possible answers |
| --- | --- | --- | --- |
| **Demographic variables** | **Demographic characteristics** | Sex | Female, male |
|  |  | Immigrant status | Native, first generation, second generation^1^ |
| **Additional control variables** | **Socioeconomic level** | Father occupation | Low, medium-low, medium, medium-high, high and unclassifiable |
|  |  | Mother occupation | Low, medium-low, medium, medium-high, high and unclassifiable |
|  |  | Does your family own a car, van or truck? | None, one, two or more |
|  |  | Do you have your own bedroom for yourself? | No, yes |
|  |  | How many computers do your family own (including laptops and tablets, not including game consoles and smartphones)? | None, one, two, more than two |
|  |  | How many bathrooms (room with a bath/shower or both) are in your home? | None, one, two, more than two |
|  |  | Does your family have a dishwasher at home? | No, yes |
|  |  | How many times did you and your family travel out of Spain for a holiday/vacation last year? | Not at all, once, twice, more than twice |
|  |  | How well-off do you think your family is? | Not at all well-off, not very well-off, average, quite well-off, very well-off |
|  | **Practice of physical activity** | Exercise - hours a week | None, half an hour, 1 hour, 2-3 hours, 4-6 hours, 7 hours or more |
|  |  | Frequency of doing vigorous physical activity | Never, less than once a month, once a week, 2-3 times a week, 4-6 times a week, every day |

Source: Authors’ own elaboration based on HBSC study.

Note: ^1^This variable is constructed from students' answers to questions about their country of birth, the country of birth of their mother and the country of birth of their father.

Table A4. Full set of parameter estimates of health and life satisfaction outcomes

|  | **Equation (2). Health and life satisfaction outcomes** | | | | | | | |
| --- | --- | --- | --- | --- | --- | --- | --- | --- |
|  | **Headache** | **Stomachache** | **Backache** | **Nervous** | **Difficulties in sleeping** | **Feeling dizzy** | **General health** | **Life satisfaction** |
| BMI | 0.234*** | 0.214*** | 0.253*** | 0.214*** | 0.205*** | 0.225*** | -0.272*** | -0.262*** |
|  | (0.017) | (0.017) | (0.014) | (0.017) | (0.022) | (0.018) | (0.013) | (0.011) |
| Female (Ref: Male) | 0.360*** | 0.407*** | 0.319*** | 0.280*** | 0.189*** | 0.221*** | -0.301*** | -0.118*** |
|  | (0.027) | (0.030) | (0.028) | (0.024) | (0.026) | (0.027) | (0.028) | (0.023) |
| Immigrant status (Ref: Native) |  |  |  |  |  |  |  |  |
| First generation | -0.055 | -0.091** | -0.108** | -0.068 | 0.093* | 0.002 | -0.007 | -0.052 |
|  | (0.047) | (0.044) | (0.046) | (0.044) | (0.050) | (0.047) | (0.052) | (0.048) |
| Second generation | 0.010 | 0.036 | 0.038 | 0.022 | 0.104** | 0.091** | -0.030 | -0.053 |
|  | (0.039) | (0.039) | (0.035) | (0.038) | (0.042) | (0.038) | (0.041) | (0.038) |
| Father occupation SES (Ref: Low) |  |  |  |  |  |  |  |  |
| Medium-Low | 0.057 | 0.039 | 0.047 | 0.062* | 0.062 | 0.018 | -0.035 | 0.007 |
|  | (0.039) | (0.041) | (0.037) | (0.036) | (0.039) | (0.041) | (0.035) | (0.035) |
| Medium | 0.061 | -0.014 | 0.046 | 0.042 | 0.058 | 0.026 | -0.059 | 0.027 |
|  | (0.057) | (0.065) | (0.059) | (0.060) | (0.071) | (0.066) | (0.060) | (0.056) |
| Medium-High | 0.006 | -0.014 | 0.034 | 0.082* | 0.021 | 0.011 | -0.041 | 0.013 |
|  | (0.049) | (0.053) | (0.051) | (0.048) | (0.055) | (0.050) | (0.051) | (0.044) |
| High | 0.102** | 0.080** | 0.086* | 0.115*** | 0.099** | 0.055 | -0.097** | -0.071* |
|  | (0.043) | (0.044) | (0.046) | (0.041) | (0.045) | (0.045) | (0.039) | (0.040) |
| Unclassifiable | 0.028 | 0.032 | 0.015 | 0.054 | 0.016 | 0.033 | -0.020 | -0.004 |
|  | (0.037) | (0.038) | (0.038) | (0.035) | (0.039) | (0.039) | (0.036) | (0.036) |
| Mother occupation SES (Ref: Low) |  |  |  |  |  |  |  |  |
| Medium-Low | 0.053 | 0.025 | 0.025 | -0.001 | 0.065 | 0.111** | -0.057 | -0.044 |
|  | (0.046) | (0.051) | (0.050) | (0.048) | (0.048) | (0.049) | (0.046) | (0.046) |
| Medium | 0.104** | 0.013 | 0.009 | 0.010 | 0.092* | 0.087* | -0.016 | -0.021 |
|  | (0.044) | (0.047) | (0.049) | (0.044) | (0.049) | (0.047) | (0.045) | (0.045) |
| Medium-High | 0.153*** | 0.043 | 0.071 | 0.023 | 0.169** | 0.113 | -0.071 | -0.095* |
|  | (0.058) | (0.064) | (0.065) | (0.061) | (0.067) | (0.069) | (0.064) | (0.057) |
| High | 0.072 | 0.048 | 0.057 | 0.017 | 0.086 | 0.113** | -0.089* | -0.073 |
|  | (0.047) | (0.052) | (0.053) | (0.053) | (0.054) | (0.053) | (0.049) | (0.046) |
| Unclassifiable | 0.099** | 0.032 | 0.018 | 0.012 | 0.063 | 0.104** | -0.019 | -0.040 |
|  | (0.039) | (0.040) | (0.041) | (0.040) | (0.043) | (0.043) | (0.039) | (0.037) |
| Family own a car, van or truck (Ref: none) |  |  |  |  |  |  |  |  |
| One | -0.099 | -0.122 | -0.068 | 0.027 | -0.107 | -0.138 | 0.141* | 0.047 |
|  | (0.080) | (0.083) | (0.082) | (0.075) | (0.087) | (0.091) | (0.080) | (0.080) |
| Two or more | -0.132 | -0.168** | -0.091 | -0.005 | -0.155* | -0.161* | 0.178** | 0.090 |
|  | (0.080) | (0.081) | (0.081) | (0.076) | (0.084) | (0.091) | (0.086) | (0.083) |
| Missing flag | -0.092 | 0.041 | 0.097 | 0.169 | -0.257 | 0.052 | 0.138 | -0.021 |
|  | (0.254) | (0.229) | (0.189) | (0.187) | (0.256) | (0.213) | (0.252) | (0.187) |
| Own bedroom (Ref: No) |  |  |  |  |  |  |  |  |
| Yes | -0.015 | -0.102*** | -0.074* | -0.022 | -0.064* | -0.068* | 0.064 | 0.018 |
|  | (0.036) | (0.037) | (0.038) | (0.037) | (0.036) | (0.040) | (0.040) | (0.035) |
| Missing flag | 0.224 | -0.092 | 0.094 | 0.026 | -0.092 | 0.057 | -0.033 | -0.092 |
|  | (0.158) | (0.174) | (0.128) | (0.146) | (0.187) | (0.166) | (0.179) | (0.124) |
| Number of computers family owned (including laptops and tablets, not including game consoles and smartphones) (Ref: none) |  |  |  |  |  |  |  |  |
| One | -0.142 | -0.200* | -0.131 | -0.227 | -0.113 | -0.030 | 0.216 | 0.101 |
|  | (0.143) | (0.121) | (0.149) | (0.139) | (0.140) | (0.132) | (0.134) | (0.134) |
| Two | -0.183 | -0.247** | -0.144 | -0.246* | -0.147 | -0.027 | 0.212* | 0.079 |
|  | (0.136) | (0.117) | (0.141) | (0.133) | (0.133) | (0.131) | (0.127) | (0.121) |
| More than 2 | -0.160 | -0.197* | -0.142 | -0.172 | -0.077 | -0.018 | 0.183 | 0.092 |
|  | (0.134) | (0.114) | (0.139) | (0.129) | (0.131) | (0.128) | (0.125) | (0.122) |
| Missing flag | -0.365* | -0.309* | -0.323 | -0.440** | -0.042 | -0.008 | 0.060 | 0.064 |
|  | (0.221) | (0.181) | (0.199) | (0.205) | (0.208) | (0.207) | (0.198) | (0.183) |
| Number of bathrooms (room with a bath/shower or both) (Ref: none) |  |  |  |  |  |  |  |  |
| One | 0.306 | 0.168 | 0.191 | 0.240 | 0.243 | 0.374 | -0.461* | -0.233 |
|  | (0.244) | (0.270) | (0.206) | (0.256) | (0.214) | (0.286) | (0.237) | (0.269) |
| Two | 0.351 | 0.232 | 0.279 | 0.259 | 0.272 | 0.368 | -0.506** | -0.236 |
|  | (0.247) | (0.273) | (0.209) | (0.256) | (0.213) | (0.288) | (0.236) | (0.269) |
| More than 2 | 0.331 | 0.247 | 0.307 | 0.265 | 0.292 | 0.423 | -0.526** | -0.240 |
|  | (0.247) | (0.272 ) | (0.208) | (0.257) | (0.220) | (0.291) | (0.237) | (0.269) |
| Missing flag | 0.553* | 0.430 | 0.512 | 0.406 | 0.458 | 0.441 | -0.259 | -0.184 |
|  | (0.313) | (0.369) | (0.326) | (0.322) | (0.304) | (0.381) | (0.346) | (0.333) |
| Availability of dishwasher at home (Ref: no) |  |  |  |  |  |  |  |  |
| Yes | 0.085** | 0.114*** | 0.096*** | 0.071* | 0.146*** | 0.117*** | -0.076** | -0.067** |
|  | (0.034) | (0.038) | (0.037) | (0.038) | (0.035) | (0.036) | (0.032) | (0.031) |
| Missing flag | 0.018 | -0.106 | -0.112 | -0.150 | 0.019 | -0.010 | 0.178 | 0.283 |
|  | (0.199) | (0.235) | (0.239) | (0.226) | (0.216) | (0.227) | (0.215) | (0.195) |
| Number of times you and your family travel out of Spain for a holiday / vacation last year (Ref: Not at all) |  |  |  |  |  |  |  |  |
| Once | 0.046 | 0.000 | 0.023 | 0.055* | 0.004 | 0.056* | 0.009 | -0.025 |
|  | (0.036) | (0.037) | (0.034) | (0.032) | (0.034) | (0.033) | (0.029) | (0.029) |
| Twice | 0.134*** | 0.172*** | 0.099** | 0.119** | 0.052 | 0.115** | -0.110** | -0.058 |
|  | (0.048) | (0.051) | (0.048) | (0.049) | (0.051) | (0.052) | (0.048) | (0.048) |
| More than twice | 0.132*** | 0.162*** | 0.102** | 0.088* | 0.176*** | 0.163*** | -0.007 | 0.012 |
|  | (0.048) | (0.049) | (0.050) | (0.049) | (0.049) | (0.056) | (0.053) | (0.048) |
| Missing flag | -0.166 | -0.053 | -0.163 | 0.169 | 0.042 | -0.229 | -0.323 | -0.190 |
|  | (0.212) | (0.208) | (0.222) | (0.214) | (0.233) | (0.275) | (0.256) | (0.240) |
| Value the well-off of the family (Ref: not at all) |  |  |  |  |  |  |  |  |
| Not very well-off | 0.168 | -0.073 | 0.062 | 0.074 | -0.042 | 0.161 | 0.043 | -0.026 |
|  | (0.147) | (0.170) | (0.177) | (0.155) | (0.150) | (0.169) | (0.159) | (0.174) |
| Average | 0.190 | 0.052 | 0.085 | 0.096 | -0.018 | 0.115 | 0.020 | 0.149 |
|  | (0.147) | (0.155) | (0.168) | (0.151) | (0.150) | (0.162) | (0.157) | (0.175) |
| Quite well-off | 0.215 | 0.126 | 0.088 | 0.122 | 0.041 | 0.225 | 0.071 | 0.180 |
|  | (0.157) | (0.165) | (0.174) | (0.157) | (0.156) | (0.169) | (0.174) | (0.185) |
| Very well-off | 0.363** | 0.274 | 0.264 | 0.153 | 0.053 | 0.333* | 0.076 | 0.281 |
|  | (0.161) | (0.171) | (0.183) | (0.169) | (0.171) | (0.172) | (0.189) | (0.200) |
| Missing flag | 0.326** | 0.235 | 0.228 | 0.145 | 0.160 | 0.192 | 0.095 | 0.248 |
|  | (0.167) | (0.184) | (0.187) | (0.182) | (0.180) | (0.188) | (0.191) | (0.205) |
| Frequency of doing vigorous physical activity (Ref: never) |  |  |  |  |  |  |  |  |
| Less than once a month | 0.051 | -0.047 | 0.014 | 0.047 | 0.141* | 0.021 | -0.012 | -0.044 |
|  | (0.069) | (0.078) | (0.073) | (0.073) | (0.076) | (0.074) | (0.069) | (0.063) |
| Once a month | -0.099 | -0.022 | -0.073 | -0.098 | 0.180** | -0.095 | 0.040 | -0.006 |
|  | (0.077) | (0.084) | (0.075) | (0.076) | (0.081) | (0.078) | (0.078) | (0.070) |
| Once a week | -0.064 | -0.041 | -0.078 | -0.049 | 0.032 | -0.090 | 0.135** | 0.050 |
|  | (0.066) | (0.073) | (0.065) | (0.066) | (0.073) | (0.069) | (0.069) | (0.059) |
| 2-3 times a week | -0.105* | -0.035 | -0.077 | -0.058 | 0.041 | -0.129** | 0.171*** | 0.120** |
|  | (0.064) | (0.070) | (0.058) | (0.065) | (0.063) | (0.066) | (0.065) | (0.052) |
| 4-6 times a week | -0.119* | -0.044 | -0.032 | -0.052 | 0.072 | -0.078 | 0.262*** | 0.089 |
|  | (0.067) | (0.072) | (0.061) | (0.065) | (0.066) | (0.065) | (0.068) | (0.055) |
| Every day | -0.055 | 0.008 | 0.009 | -0.032 | 0.079 | -0.046 | 0.294*** | 0.147** |
|  | (0.076) | (0.076) | (0.062) | (0.073) | (0.071) | (0.077) | (0.078) | (0.063) |
| Missing flag | -0.038 | 0.209** | 0.013 | 0.088 | 0.246** | 0.063 | 0.162 | -0.014 |
|  | (0.115) | (0.118) | (0.113) | (0.111) | (0.112) | (0.103) | (0.102) | (0.102) |
| Exercise - hours a week (Ref: none) |  |  |  |  |  |  |  |  |
| Half an hour | 0.034 | -0.002 | -0.024 | -0.015 | -0.014 | 0.024 | -0.006 | 0.074 |
|  | (0.055) | (0.058) | (0.053) | (0.054) | (0.058) | (0.058) | (0.052) | (0.048) |
| 1 hour | -0.048 | -0.090** | -0.037 | -0.044 | -0.052 | -0.032 | 0.032 | 0.090** |
|  | (0.050) | (0.051) | (0.049) | (0.045) | (0.048) | (0.051) | (0.047) | (0.039) |
| 2-3 hours | -0.026 | -0.092** | -0.022 | -0.068 | -0.066 | -0.040 | 0.028 | 0.047 |
|  | (0.049) | (0.049) | (0.046) | (0.044) | (0.046) | (0.051) | (0.048) | (0.043) |
| 4-6 hours | -0.115** | -0.184*** | -0.139*** | -0.109** | -0.104** | -0.120** | 0.118** | 0.113** |
|  | (0.057) | (0.054) | (0.053) | (0.050) | (0.052) | (0.055) | (0.054) | (0.046) |
| 7 hours or more | -0.028 | -0.076 | 0.017 | -0.012 | 0.000 | -0.013 | 0.173*** | 0.049 |
|  | (0.063) | (0.064) | (0.058) | (0.063) | (0.064) | (0.064) | (0.062) | (0.051) |
| Missing flag | -0.001 | -0.279*** | -0.070 | -0.107 | -0.199* | -0.081 | 0.039 | 0.086 |
|  | (0.105) | (0.104) | (0.115) | (0.101) | (0.105) | (0.101) | (0.099) | (0.098) |
| Constant cut1 | 5.398*** | 4.626*** | 5.556*** | 4.440*** | 4.783*** | 5.420*** | -7.344*** | -6.741*** |
|  | (0.462) | (0.511) | (0.434) | (0.520) | (0.563) | (0.484) | (0.359) | (0.407) |
| Constant cut2 | 5.720*** | 5.131*** | 5.864*** | 4.861*** | 5.075*** | 5.744*** | -6.631*** | -6.640*** |
|  | (0.445) | (0.486) | (0.417) | (0.499) | (0.544) | (0.466) | (0.383) | (0.409) |
| Constant cut3 | 5.907*** | 5.314*** | 6.055*** | 5.140*** | 5.270*** | 5.940*** | -5.408*** | -6.539*** |
|  | (0.435) | (0.477) | (0.405) | (0.485) | (0.532) | (0.455) | (0.447) | (0.412) |
| Constant cut4 | 6.413*** | 5.754*** | 6.344*** | 5.515*** | 5.573*** | 6.278*** |  | -6.413*** |
|  | (0.410) | (0.455) | (0.390) | (0.468) | (0.515) | (0.439) |  | (0.415) |
| Constant cut5 |  |  |  |  |  |  |  | -6.250*** |
|  |  |  |  |  |  |  |  | (0.422) |
| Constant cut6 |  |  |  |  |  |  |  | -5.951*** |
|  |  |  |  |  |  |  |  | (0.434) |
| Constant cut7 |  |  |  |  |  |  |  | -5.745*** |
|  |  |  |  |  |  |  |  | (0.443) |
| Constant cut8 |  |  |  |  |  |  |  | -5.429*** |
|  |  |  |  |  |  |  |  | (0.457) |
| Constant cut9 |  |  |  |  |  |  |  | -5.023*** |
|  |  |  |  |  |  |  |  | (0.476) |
| Constant cut10 |  |  |  |  |  |  |  | -4.627*** |
|  |  |  |  |  |  |  |  | (0.495) |
| **Equation (3). BMI equation** | | | | | | | | |
| Alcohol consumption (Ref: Never) |  |  |  |  |  |  |  |  |
| 1-2 days | 0.963*** | 0.971*** | 0.998*** | 1.012*** | 0.901*** | 0.913*** | 0.950*** | 0.994*** |
|  | (0.107) | (0.106) | (0.105) | (0.107) | (0.119) | (0.110) | (0.107) | (0.105) |
| 3-5 days | 1.167*** | 1.064*** | 1.112*** | 1.074*** | 1.129*** | 1.159*** | 1.002*** | 1.091*** |
|  | (0.143) | (0.155) | (0.133) | (0.146) | (0.153) | (0.143) | (0.158) | (0.132) |
| 6-9 days | 1.114*** | 1.211*** | 1.093*** | 1.027*** | 1.120*** | 1.109*** | 1.063*** | 0.973*** |
|  | (0.194) | (0.181) | (0.176) | (0.189) | (0.194) | (0.177) | (0.180) | (0.158) |
| 10-19 days | 1.677*** | 1.793*** | 1.514*** | 1.491*** | 1.875*** | 1.967*** | 1.887*** | 1.397*** |
|  | (0.416) | (0.398) | (0.372) | (0.409) | (0.428) | (0.386) | (0.372) | (0.340) |
| 20-29 days | 0.834 | 1.357** | 1.097 | 1.523** | 1.218* | 1.493** | 1.833*** | 0.569 |
|  | (0.669) | (0.672) | (0.697) | (0.619) | (0.726) | (0.682) | (0.516) | (0.375) |
| 30 days (or more) | 0.638** | 0.484 | 0.613* | 0.957*** | 1.102*** | 0.558* | 0.806** | 0.916*** |
|  | (0.320) | (0.371) | (0.343) | (0.372) | (0.379) | (0.311) | (0.359) | (0.336) |
| Missing flag | 0.320** | 0.404*** | 0.512*** | 0.468*** | 0.467*** | 0.426*** | 0.622*** | 0.619*** |
|  | (0.147) | (0.148) | (0.131) | (0.146) | (0.155) | (0.146) | (0.134) | (0.123) |
| Female (Ref: Male) | -0.344*** | -0.343*** | -0.344*** | -0.345*** | -0.342*** | -0.343*** | -0.342*** | -0.344*** |
|  | (0.075) | (0.075) | (0.075) | (0.075) | (0.075) | (0.075) | (0.075) | (0.075) |
| Immigrant status (Ref: Native) |  |  |  |  |  |  |  |  |
| First generation | 0.315*** | 0.313** | 0.310** | 0.311** | 0.311** | 0.312** | 0.306** | 0.308** |
|  | (0.144) | (0.143) | (0.144) | (0.144) | (0.143) | (0.144) | (0.143) | (0.143) |
| Second generation | -0.052 | -0.053 | -0.054 | -0.054 | -0.052 | -0.053 | -0.055 | -0.055 |
|  | (0.119) | (0.119) | (0.119) | (0.119) | (0.119) | (0.119) | (0.119) | (0.119) |
| Father occupation SES (Ref: Low) |  |  |  |  |  |  |  |  |
| Medium-Low | -0.117 | -0.118 | -0.117 | -0.118 | -0.120 | -0.119 | -0.119 | -0.115 |
|  | (0.107) | (0.107) | (0.106) | (0.107) | (0.106) | (0.106) | (0.106) | (0.106) |
| Medium | -0.147 | -0.147 | -0.151 | -0.152 | -0.148 | -0.147 | -0.151 | -0.153 |
|  | (0.183) | (0.183) | (0.183) | (0.183) | (0.183) | (0.183) | (0.183) | (0.183) |
| Medium-High | -0.085 | -0.084 | -0.085 | -0.086 | -0.087 | -0.086 | -0.086 | -0.085 |
|  | (0.148) | (0.148) | (0.148) | (0.148) | (0.148) | (0.148) | (0.148) | (0.148) |
| High | -0.469*** | -0.469*** | -0.469*** | -0.472*** | -0.473*** | -0.470*** | -0.471*** | -0.470*** |
|  | (0.124) | (0.124) | (0.124) | (0.124) | (0.124) | (0.124) | (0.124) | (0.123) |
| Unclassifiable | 0.012 | 0.012 | 0.012 | 0.010 | 0.009 | 0.011 | 0.011 | 0.011 |
|  | (0.109) | (0.109) | (0.109) | (0.109) | (0.109) | (0.109) | (0.109) | (0.109) |
| Mother occupation SES (Ref: Low) |  |  |  |  |  |  |  |  |
| Medium-Low | -0.362** | -0.364** | -0.364** | -0.365** | -0.366*** | -0.366*** | -0.370*** | -0.365** |
|  | (0.143) | (0.143) | (0.143) | (0.143) | (0.143) | (0.143) | (0.143) | (0.143) |
| Medium | -0.302** | -0.304** | -0.305** | -0.305** | -0.304** | -0.306** | -0.309** | -0.305** |
|  | (0.131) | (0.131) | (0.131) | (0.131) | (0.131) | (0.131) | (0.131) | (0.131) |
| Medium-High | -0.497*** | -0.501*** | -0.502*** | -0.503*** | -0.502*** | -0.500*** | -0.507*** | -0.503*** |
|  | (0.177) | (0.177) | (0.177) | (0.177) | (0.177) | (0.177) | (0.177) | (0.177) |
| High | -0.526*** | -0.529*** | -0.527*** | -0.527*** | -0.527*** | -0.529*** | -0.531*** | -0.526*** |
|  | (0.139) | (0.139) | (0.139) | (0.139) | (0.139) | (0.139) | (0.139) | (0.138) |
| Unclassifiable | -0.227* | -0.230* | -0.228* | -0.229* | -0.231* | -0.232* | -0.235** | -0.228* |
|  | (0.119) | (0.119) | (0.119) | (0.119) | (0.119) | (0.119) | (0.119) | (0.119) |
| Family own a car, van or truck (Ref: none) |  |  |  |  |  |  |  |  |
| One | 0.316 | 0.315 | 0.315 | 0.318 | 0.317 | 0.314 | 0.317 | 0.319 |
|  | (0.245) | (0.245) | (0.245) | (0.245) | (0.245) | (0.245) | (0.245) | (0.245) |
| Two or more | 0.388 | 0.387 | 0.386 | 0.389 | 0.390 | 0.387 | 0.389 | 0.389 |
|  | (0.255) | (0.255) | (0.255) | (0.255) | (0.255) | (0.255) | (0.255) | (0.255) |
| Missing flag | -0.087 | -0.093 | -0.090 | -0.084 | -0.077 | -0.088 | -0.086 | -0.083 |
|  | (0.603) | (0.602) | (0.601) | (0.603) | (0.603) | (0.602) | (0.603) | (0.602) |
| Own bedroom (Ref: No) |  |  |  |  |  |  |  |  |
| Yes | 0.199* | 0.201* | 0.202* | 0.202* | 0.204* | 0.201* | 0.208* | 0.205* |
|  | (0.117) | (0.117) | (0.116) | (0.116) | (0.117) | (0.117) | (0.117) | (0.116) |
| Missing flag | -0.562* | -0.556* | -0.546* | -0.551* | -0.560* | -0.558* | -0.543* | -0.538* |
|  | (0.322) | (0.324) | (0.322) | (0.322) | (0.325) | (0.324) | (0.325) | (0.323) |
| Number of computers family owned (including laptops and tablets, not including game consoles and smartphones) (Ref: none) |  |  |  |  |  |  |  |  |
| One | 0.151 | 0.152 | 0.153 | 0.149 | 0.154 | 0.155 | 0.154 | 0.154 |
|  | (0.430) | (0.429) | (0.429) | (0.429) | (0.429) | (0.430) | (0.428) | (0.428) |
| Two | 0.162 | 0.165 | 0.166 | 0.160 | 0.168 | 0.169 | 0.169 | 0.168 |
|  | (0.405) | (0.405) | (0.405) | (0.405) | (0.406) | (0.406) | (0.405) | (0.404) |
| More than 2 | 0.225 | 0.226 | 0.227 | 0.220 | 0.229 | 0.231 | 0.227 | 0.227 |
|  | (0.398) | (0.398) | (0.398) | (0.398) | (0.398) | (0.399) | (0.397) | (0.397) |
| Missing flag | 0.296 | 0.291 | 0.300 | 0.298 | 0.298 | 0.295 | 0.293 | 0.304 |
|  | (0.598) | (0.598) | (0.598) | (0.597) | (0.599) | (0.599) | (0.598) | (0.597) |
| Number of bathrooms (room with a bath/shower or both) (Ref: none) |  |  |  |  |  |  |  |  |
| One | -0.800 | -0.792 | -0.788 | -0.795 | -0.790 | -0.791 | -0.780 | -0.782 |
|  | (0.821) | (0.821) | (0.817) | (0.818) | (0.817) | (0.819) | (0.814) | (0.814) |
| Two | -1.008 | -0.999 | -0.995 | -1.001 | -0.994 | -0.998 | -0.984 | -0.987 |
|  | (0.828) | (0.827) | (0.823) | (0.824) | (0.823) | (0.825) | (0.820) | (0.820) |
| More than 2 | -1.011 | -1.001 | -0.997 | -1.004 | -0.999 | -1.002 | -0.988 | -0.991 |
|  | (0.830) | (0.829) | (0.826) | (0.827) | (0.825) | (0.827) | (0.823) | (0.823) |
| Missing flag | -0.838 | -0.833 | -0.831 | -0.846 | -0.832 | -0.822 | -0.824 | -0.830 |
|  | (1.031) | (1.030) | (1.027) | (1.029) | (1.028) | (1.029) | (1.026) | (1.026) |
| Availability of dishwasher at home (Ref: no) |  |  |  |  |  |  |  |  |
| Yes | -0.460*** | -0.460*** | -0.458*** | -0.459*** | -0.461*** | -0.460*** | -0.459*** | -0.457*** |
|  | (0.100) | (0.100) | (0.100) | (0.100) | (0.100) | (0.100) | (0.100) | (0.100) |
| Missing flag | -0.225 | -0.217 | -0.223 | -0.229 | -0.236 | -0.225 | -0.226 | -0.232 |
|  | (0.685) | (0.686) | (0.685) | (0.687) | (0.687) | (0.685) | (0.688) | (0.687) |
| Number of times you and your family travel out of Spain for a holiday / vacation last year (Ref: Not at all) |  |  |  |  |  |  |  |  |
| Once | -0.278*** | -0.278*** | -0.278*** | -0.278*** | -0.278*** | -0.279*** | -0.279*** | -0.279*** |
|  | (0.094) | (0.094) | (0.094) | (0.094) | (0.095) | (0.095) | (0.094) | (0.094) |
| Twice | -0.573*** | -0.573*** | -0.575*** | -0.574*** | -0.576*** | -0.574*** | -0.577*** | -0.578*** |
|  | (0.130) | (0.130) | (0.130) | (0.130) | (0.130) | (0.130) | (0.130) | (0.130) |
| More than twice | -0.528*** | -0.533*** | -0.530*** | -0.532*** | -0.535*** | -0.534*** | -0.539*** | -0.530*** |
|  | (0.156) | (0.156) | (0.156) | (0.155) | (0.156) | (0.156) | (0.155) | (0.156) |
| Missing flag | 0.513 | 0.513 | 0.504 | 0.514 | 0.521 | 0.508 | 0.512 | 0.506 |
|  | (0.709) | (0.712) | (0.713) | (0.713) | (0.713) | (0.711) | (0.718) | (0.715) |
| Value the well-off of the family (Ref: not at all) |  |  |  |  |  |  |  |  |
| Not very well-off | -0.779 | -0.771 | -0.777 | -0.777 | -0.772 | -0.775 | -0.766 | -0.776 |
|  | (0.612) | (0.612) | (0.613) | (0.614) | (0.611) | (0.612) | (0.612) | (0.616) |
| Average | -1.548*** | -1.540*** | -1.542*** | -1.541*** | -1.540*** | -1.545*** | -1.533*** | -1.540*** |
|  | (0.578) | (0.578) | (0.580) | (0.580) | (0.577) | (0.577) | (0.578) | (0.582) |
| Quite well-off | -1.758*** | -1.752*** | -1.754*** | -1.753*** | -1.759*** | -1.759*** | -1.751*** | -1.755*** |
|  | (0.609) | (0.609) | (0.610) | (0.611) | (0.608) | (0.608) | (0.608) | (0.612) |
| Very well-off | -1.843*** | -1.837*** | -1.843*** | -1.843*** | -1.842*** | -1.839*** | -1.836*** | -1.847*** |
|  | (0.622) | (0.622) | (0.623) | (0.623) | (0.621) | (0.620) | (0.622) | (0.625) |
| Missing flag | -1.879*** | -1.872*** | -1.880*** | -1.880*** | -1.876*** | -1.877*** | -1.874*** | -1.885*** |
|  | (0.637) | (0.637) | (0.638) | (0.638) | (0.636) | (0.636) | (0.636) | (0.640) |
| Frequency of doing vigorous physical activity (Ref: never) |  |  |  |  |  |  |  |  |
| Less than once a month | -0.006 | -0.009 | -0.007 | -0.005 | -0.001 | -0.007 | -0.006 | -0.001 |
|  | (0.226) | (0.226) | (0.226) | (0.226) | (0.226) | (0.226) | (0.226) | (0.226) |
| Once a month | 0.236 | 0.231 | 0.229 | 0.232 | 0.231 | 0.232 | 0.225 | 0.227 |
|  | (0.244) | (0.244) | (0.245) | (0.244) | (0.245) | (0.245) | (0.245) | (0.245) |
| Once a week | 0.418** | 0.414** | 0.416** | 0.418** | 0.419** | 0.416** | 0.415** | 0.420** |
|  | (0.194) | (0.195) | (0.195) | (0.194) | (0.194) | (0.194) | (0.195) | (0.195) |
| 2-3 times a week | 0.182 | 0.181 | 0.181 | 0.184 | 0.187 | 0.184 | 0.184 | 0.182 |
|  | (0.185) | (0.185) | (0.185) | (0.185) | (0.185) | (0.185) | (0.185) | (0.185) |
| 4-6 times a week | 0.086 | 0.086 | 0.087 | 0.089 | 0.091 | 0.090 | 0.091 | 0.089 |
|  | (0.203) | (0.203) | (0.203) | (0.203) | (0.203) | (0.203) | (0.204) | (0.203) |
| Every day | -0.303 | -0.303 | -0.304 | -0.304 | -0.302 | -0.303 | -0.305 | -0.306 |
|  | (0.195) | (0.195) | (0.195) | (0.195) | (0.195) | (0.195) | (0.195) | (0.195) |
| Missing flag | -0.096 | -0.100 | -0.103 | -0.099 | -0.100 | -0.100 | -0.106 | -0.106 |
|  | (0.300) | (0.300) | (0.300) | (0.300) | (0.300) | (0.300) | (0.300) | (0.300) |
| Exercise - hours a week (Ref: none) |  |  |  |  |  |  |  |  |
| Half an hour | 0.173 | 0.171 | 0.173 | 0.172 | 0.173 | 0.172 | 0.170 | 0.175 |
|  | (0.152) | (0.152) | (0.152) | (0.152) | (0.152) | (0.152) | (0.152) | (0.152) |
| 1 hour | 0.233* | 0.233** | 0.233* | 0.231* | 0.232* | 0.232* | 0.232* | 0.235* |
|  | (0.130) | (0.130) | (0.130) | (0.130) | (0.130) | (0.130) | (0.130) | (0.130) |
| 2-3 hours | 0.178 | 0.176 | 0.176 | 0.175 | 0.177 | 0.176 | 0.174 | 0.178 |
|  | (0.128) | (0.128) | (0.128) | (0.128) | (0.128) | (0.128) | (0.128) | (0.128) |
| 4-6 hours | 0.363** | 0.362** | 0.360** | 0.360** | 0.365** | 0.364** | 0.362** | 0.361** |
|  | (0.155) | (0.155) | (0.155) | (0.156) | (0.155) | (0.155) | (0.155) | (0.155) |
| 7 hours or more | 0.159 | 0.157 | 0.156 | 0.155 | 0.158 | 0.158 | 0.156 | 0.158 |
|  | (0.163) | (0.163) | (0.163) | (0.163) | (0.163) | (0.163) | (0.163) | (0.163) |
| Missing flag | 0.308 | 0.309 | 0.311 | 0.311 | 0.313 | 0.310 | 0.316 | 0.316 |
|  | (0.301) | (0.301) | (0.301) | (0.301) | (0.301) | (0.301) | (0.301) | (0.301) |
| Constant | 22.223*** | 22.208*** | 22.198*** | 22.209*** | 22.196*** | 22.208*** | 22.178*** | 22.180*** |
|  | (1.094) | ( 1.094) | (1.092) | (1.095) | (1.092) | (1.094) | (1.091) | (1.093) |
|  |  |  |  |  |  |  |  |  |
| Observations | 9369 | 9363 | 9341 | 9338 | 9335 | 9316 | 9487 | 9435 |
| *F statistic* of Stock and Yogo (2005) test of weak instruments | 58.173*** | 50.914*** | 60.305*** | 88.779*** | 35.199*** | 51.474*** | 92.779*** | 114.393*** |

Dependent variable: ordinal dependent variables in Equation (2) and Body Mass Index (continuous variable) in Equation (3).

Method of estimation: conditional (recursive) mixed process estimator. Ordered probit model employed in Equation (2) and OLS in Equation (3). Standard errors are in parentheses and are clustered at school level. In equation (2), coefficients referred to probit coefficients. Full estimates of marginal effects are available upon request to authors.

The null hypothesis of the Stock and Yogo test (2005) is that the set of instrument is weak.

Coefficient: ***significant at 1%, ** at 5%, * at 10%.

Source: Authors’ own calculations.

Table A5. Full set of parameter estimates of fighting and bullying outcomes

| **Equation (2). Fighting and bullying outcomes** | | | | | |
| --- | --- | --- | --- | --- | --- |
|  | **Physical fight** | **Bullying others** | **Been bullied** | **Cyberbullied by messages** | **Cyberbullied by pictures** |
| BMI | 0.202*** | 0.217*** | -0.079** | 0.173*** | 0.192*** |
|  | (0.026) | (0.019) | (0.043) | (0.035) | (0.027) |
| Female (Ref: Male) | -0.335*** | -0.134*** | -0.273*** | -0.148*** | -0.121*** |
|  | (0.057) | (0.037) | (0.036) | (0.049) | (0.047) |
| Immigrant status (Ref: Native) |  |  |  |  |  |
| First generation | 0.076 | 0.039 | 0.234*** | 0.109* | 0.045 |
|  | (0.058) | (0.050) | (0.054) | (0.065) | (0.062) |
| Second generation | 0.135*** | 0.107** | 0.217*** | 0.044 | 0.031 |
|  | (0.046) | (0.046) | (0.056) | (0.059) | (0.058) |
| Father occupation SES (Ref: Low) |  |  |  |  |  |
| Medium-Low | 0.055 | 0.047 | 0.024 | 0.007 | 0.051 |
|  | (0.044) | (0.045) | (0.055) | (0.057) | (0.063) |
| Medium | -0.105 | 0.047 | -0.066 | -0.036 | -0.030 |
|  | (0.083) | (0.070) | (0.091) | (0.095) | (0.097) |
| Medium-High | 0.000 | -0.047 | 0.005 | -0.067 | -0.003 |
|  | (0.057) | (0.060) | (0.080) | (0.083) | (0.080) |
| High | 0.054 | 0.036 | -0.056 | 0.035 | 0.064 |
|  | (0.056) | (0.055) | (0.068) | (0.072) | (0.071) |
| Unclassifiable | -0.022 | -0.038 | -0.033 | -0.034 | -0.053 |
|  | (0.046) | (0.044) | (0.049) | (0.057) | (0.059) |
| Mother occupation SES (Ref: Low) |  |  |  |  |  |
| Medium-Low | 0.068 | -0.028 | -0.112** | 0.081 | 0.036 |
|  | (0.057) | (0.054) | (0.061) | (0.069) | (0.070) |
| Medium | -0.009 | -0.020 | -0.124** | -0.015 | 0.017 |
|  | (0.054) | (0.053) | (0.067) | (0.073) | (0.070) |
| Medium-High | 0.003 | 0.049 | -0.140* | 0.051 | 0.111 |
|  | (0.077) | (0.077) | (0.083) | (0.098) | (0.091) |
| High | -0.032 | 0.003 | -0.174*** | -0.076 | 0.045 |
|  | (0.062) | (0.060) | (0.065) | (0.088) | (0.068) |
| Unclassifiable | -0.040 | -0.034 | -0.105* | 0.045 | 0.047 |
|  | (0.049) | (0.043) | (0.054) | (0.061) | (0.061) |
| Family own a car, van or truck (Ref: none) |  |  |  |  |  |
| One | -0.124 | -0.091 | -0.009 | -0.016 | -0.220* |
|  | (0.087) | (0.097) | (0.103) | (0.114) | (0.124) |
| Two or more | -0.093 | -0.122 | 0.002 | -0.044 | -0.206 |
|  | (0.094) | (0.101) | (0.099) | (0.119) | (0.126) |
| Missing flag | 0.096 | -0.419 | -0.389 | -0.467 | -0.769 |
|  | (0.281) | (0.324) | (0.450) | (0.518) | (0.574) |
| Own bedroom (Ref: No) |  |  |  |  |  |
| Yes | -0.193*** | -0.067 | -0.016 | 0.015 | -0.038 |
|  | (0.039) | (0.045) | (0.052) | (0.066) | (0.062) |
| Missing flag | 0.355** | 0.324* | 0.106 | 0.200 | 0.398* |
|  | (0.169) | (0.170) | (0.278) | (0.304) | (0.222) |
| Number of computers family owned (including laptops and tablets, not including game consoles and smartphones) (Ref: none) |  |  |  |  |  |
| One | -0.151 | -0.098 | -0.007 | -0.098 | -0.243 |
|  | (0.148) | (0.124) | (0.155) | (0.204) | (0.186) |
| Two | -0.183 | -0.059 | -0.108 | -0.243 | -0.365* |
|  | (0.147) | (0.124) | (0.152) | (0.203) | (0.187) |
| More than 2 | -0.162 | -0.037 | -0.043 | -0.234 | -0.357* |
|  | (0.146) | (0.115) | (0.150) | (0.205) | (0.184) |
| Missing flag | -0.159 | 0.122 | 0.250 | -0.018 | -0.319 |
|  | (0.219) | (0.210) | (0.268) | (0.288) | (0.319) |
| Number of bathrooms (room with a bath/shower or both) (Ref: none) |  |  |  |  |  |
| One | 0.144 | 0.001 | -0.414 | 0.090 | -0.107 |
|  | (0.246) | (0.277) | (0.275) | (0.298) | (0.275) |
| Two | 0.123 | 0.045 | -0.450* | 0.095 | -0.109 |
|  | (0.244) | (0.275) | (0.273) | (0.296) | (0.271) |
| More than 2 | 0.235 | 0.136 | -0.390 | 0.115 | -0.114 |
|  | (0.249) | (0.284) | (0.279) | (0.305) | (0.283) |
| Missing flag | -0.154 | 0.148 | -0.558 | 0.069 | -0.008 |
|  | (0.361) | (0.413) | (0.450) | (0.509) | (0.446) |
| Availability of dishwasher at home (Ref: no) |  |  |  |  |  |
| Yes | 0.069* | 0.060 | -0.081 | 0.073 | 0.137** |
|  | (0.041) | (0.043) | (0.051) | (0.053) | (0.054) |
| Missing flag | 0.126 | -0.425 | -0.348 | -0.432 | -0.104 |
|  | (0.213) | (0.304) | (0.415) | (0.524) | (0.409) |
| Number of times you and your family travel out of Spain for a holiday / vacation last year (Ref: Not at all) |  |  |  |  |  |
| Once | 0.049 | 0.025 | 0.003 | 0.015 | 0.031 |
|  | (0.038) | (0.045) | (0.045) | (0.052) | (0.052) |
| Twice | 0.216*** | 0.148*** | -0.001 | 0.227*** | 0.174** |
|  | (0.051) | (0.057) | (0.072) | (0.070) | (0.077) |
| More than twice | 0.263*** | 0.189*** | 0.021 | 0.204** | 0.263*** |
|  | (0.052) | (0.062) | (0.085) | (0.080) | (0.074) |
| Missing flag | 0.437 | 0.284 | 0.527* | 0.602*** | 0.322 |
|  | (0.297) | (0.274) | (0.316) | (0.228) | (0.284) |
| Value the well-off of the family (Ref: not at all) |  |  |  |  |  |
| Not very well-off | 0.018 | -0.027 | -0.357* | -0.106 | 0.035 |
|  | (0.166) | (0.152) | (0.193) | (0.166) | (0.174) |
| Average | 0.013 | 0.125 | -0.693*** | -0.078 | 0.040 |
|  | (0.171) | (0.138) | (0.181) | (0.172) | (0.173) |
| Quite well-off | 0.174 | 0.251* | -0.610*** | 0.120 | 0.175 |
|  | (0.177) | (0.150) | (0.195) | (0.174) | (0.181) |
| Very well-off | 0.217 | 0.277* | -0.575*** | 0.381** | 0.490*** |
|  | (0.200) | (0.165) | (0.215) | (0.180) | (0.190) |
| Missing flag | -0.522 | 0.172 | -0.656*** | 0.279 | 0.180 |
|  | (0.381) | (0.182) | (0.236) | (0.210) | (0.238) |
| Frequency of doing vigorous physical activity (Ref: never) |  |  |  |  |  |
| Less than once a month | 0.082 | 0.129 | 0.054 | 0.120 | 0.055 |
|  | (0.086) | (0.090) | (0.095) | (0.101) | (0.105) |
| Once a month | 0.093 | 0.142 | 0.080 | 0.098 | 0.158 |
|  | (0.098) | (0.091) | (0.118) | (0.113) | (0.113) |
| Once a week | -0.080 | 0.040 | 0.034 | 0.009 | 0.035 |
|  | (0.076) | (0.077) | (0.095) | (0.095) | (0.098) |
| 2-3 times a week | -0.043 | 0.048 | 0.036 | 0.019 | 0.046 |
|  | (0.067) | (0.074) | (0.084) | (0.088) | (0.089) |
| 4-6 times a week | 0.003 | 0.050 | -0.091 | 0.125 | 0.102 |
|  | (0.073) | (0.074) | (0.094) | (0.093) | (0.099) |
| Every day | 0.160** | 0.210*** | -0.015 | 0.227** | 0.275*** |
|  | (0.071) | (0.081) | (0.096) | (0.092) | (0.093) |
| Missing flag | 0.119 | 0.180 | 0.084 | 0.075 | 0.162 |
|  | (0.140) | (0.128) | (0.156) | (0.183) | (0.189) |
| Exercise - hours a week (Ref: none) |  |  |  |  |  |
| Half an hour | 0.000 | -0.056 | 0.094 | -0.039 | -0.056 |
|  | (0.065) | (0.060) | (0.073) | (0.081) | (0.077) |
| 1 hour | -0.073 | -0.129** | 0.023 | -0.130* | -0.108 |
|  | (0.052) | (0.057) | (0.066) | (0.066) | (0.069) |
| 2-3 hours | -0.066 | -0.117** | -0.012 | -0.209*** | -0.132** |
|  | (0.056) | (0.056) | (0.067) | (0.067) | (0.065) |
| 4-6 hours | -0.133** | -0.203*** | -0.022 | -0.336*** | -0.188** |
|  | (0.061) | (0.067) | (0.079) | (0.076) | (0.078) |
| 7 hours or more | -0.076 | -0.139** | -0.103 | -0.232*** | -0.161* |
|  | (0.071) | (0.074) | (0.085) | (0.089) | (0.091) |
| Missing flag | -0.053 | -0.120 | 0.079 | -0.063 | -0.161 |
|  | (0.132) | (0.117) | (0.151) | (0.175) | (0.191) |
| Constant cut1 | 4.105*** | 4.999*** | -1.925* | 4.460*** | 4.515*** |
|  | (0.692) | (0.529) | (1.038) | (0.820) | (0.703) |
| Constant cut2 | 4.536*** | 5.538*** | -1.326 | 4.741*** | 4.802*** |
|  | (0.657) | (0.497) | (1.057) | (0.800) | (0.684) |
| Constant cut3 | 4.838*** | 5.802*** | -1.040 | 4.976*** | 5.032*** |
|  | (0.634) | (0.483) | (1.066) | (0.782) | (0.668) |
| Constant cut4 | 5.051*** | 6.025*** | -0.810 | 5.183*** | 5.205*** |
|  | (0.617) | (0.472) | (1.073) | (0.769) | (0.658) |
| **Equation (3). BMI equation** | | | | | |
| Alcohol consumption (Ref: Never) |  |  |  |  |  |
| 1-2 days | 0.899*** | 0.911*** | 0.999*** | 0.931*** | 0.945*** |
|  | (0.122) | (0.113) | (0.105) | (0.117) | (0.110) |
| 3-5 days | 1.082*** | 1.053*** | 1.088*** | 1.146*** | 1.135*** |
|  | (0.164) | (0.165) | (0.184) | (0.168) | (0.174) |
| 6-9 days | 1.176*** | 1.078*** | 0.981*** | 0.910*** | 1.016*** |
|  | (0.218) | (0.213) | (0.232) | (0.222) | (0.209) |
| 10-19 days | 1.855*** | 2.003*** | 1.820*** | 1.930*** | 1.916*** |
|  | (0.440) | (0.398) | (0.487) | (0.451) | (0.441) |
| 20-29 days | 1.921*** | 1.889*** | 1.840* | 1.808** | 1.567* |
|  | (0.732) | (0.591) | (1.029) | (0.858) | (0.910) |
| 30 days (or more) | 0.882** | 0.769** | 0.475 | 0.797** | 0.589* |
|  | (0.390) | (0.364) | (0.377) | (0.377) | (0.353) |
| Missing flag | 0.545*** | 0.591*** | 0.416** | 0.538*** | 0.509*** |
|  | (0.163) | (0.153) | (0.185) | (0.173) | (0.162) |
| Female (Ref: Male) | -0.342*** | -0.342*** | -0.344*** | -0.343*** | -0.343*** |
|  | (0.075) | (0.075) | (0.075) | (0.075) | (0.075) |
| Immigrant status (Ref: Native) |  |  |  |  |  |
| First generation | 0.309** | 0.307** | 0.311** | 0.308** | 0.309** |
|  | (0.144) | (0.143) | (0.144) | (0.144) | (0.144) |
| Second generation | -0.054 | -0.054 | -0.054 | -0.054 | -0.054 |
|  | (0.119) | (0.119) | (0.119) | (0.118) | (0.119) |
| Father occupation SES (Ref: Low) |  |  |  |  |  |
| Medium-Low | -0.121 | -0.120 | -0.118 | -0.119 | -0.118 |
|  | (0.106) | (0.106) | (0.107) | (0.106) | (0.107) |
| Medium | -0.149 | -0.149 | -0.150 | -0.152 | -0.150 |
|  | (0.183) | (0.183) | (0.183) | (0.183) | (0.183) |
| Medium-High | -0.087 | -0.086 | -0.085 | -0.086 | -0.085 |
|  | (0.148) | (0.148) | (0.148) | (0.148) | (0.148) |
| High | -0.473*** | -0.471*** | -0.469*** | -0.471*** | -0.469*** |
|  | (0.124) | (0.124) | (0.124) | (0.124) | (0.124) |
| Unclassifiable | 0.009 | 0.010 | 0.012 | 0.010 | 0.011 |
|  | (0.109) | (0.109) | (0.109) | (0.109) | (0.109) |
| Mother occupation SES (Ref: Low) |  |  |  |  |  |
| Medium-Low | -0.367*** | -0.369*** | -0.366*** | -0.369*** | -0.368*** |
|  | (0.143) | (0.143) | (0.143) | (0.143) | (0.143) |
| Medium | -0.306** | -0.309** | -0.307** | -0.309** | -0.308** |
|  | (0.131) | (0.131) | (0.130) | (0.131) | (0.130) |
| Medium-High | -0.505*** | -0.506*** | -0.502*** | -0.503*** | -0.502*** |
|  | (0.177) | (0.177) | (0.177) | (0.177) | (0.177) |
| High | -0.530*** | -0.531*** | -0.530*** | -0.530*** | -0.530*** |
|  | (0.139) | (0.139) | (0.139) | (0.139) | (0.139) |
| Unclassifiable | -0.233** | -0.235** | -0.232* | -0.234** | -0.233** |
|  | (0.119) | (0.119) | (0.119) | (0.119) | (0.119) |
| Family own a car, van or truck (Ref: none) |  |  |  |  |  |
| One | 0.315 | 0.316 | 0.316 | 0.317 | 0.315 |
|  | (0.245) | (0.245) | (0.245) | (0.245) | (0.245) |
| Two or more | 0.387 | 0.389 | 0.388 | 0.388 | 0.387 |
|  | (0.255) | (0.255) | (0.255) | (0.255) | (0.255) |
| Missing flag | -0.085 | -0.086 | -0.092 | -0.083 | -0.087 |
|  | (0.603) | (0.602) | (0.603) | (0.602) | (0.602) |
| Own bedroom (Ref: No) |  |  |  |  |  |
| Yes | 0.205* | 0.207* | 0.201* | 0.204* | 0.203* |
|  | (0.117) | (0.117) | (0.117) | (0.117) | (0.117) |
| Missing flag | -0.551* | -0.547* | -0.553* | -0.550* | -0.551* |
|  | (0.325) | (0.325) | (0.324) | (0.325) | (0.324) |
| Number of computers family owned (including laptops and tablets, not including game consoles and smartphones) (Ref: none) |  |  |  |  |  |
| One | 0.155 | 0.156 | 0.151 | 0.155 | 0.155 |
|  | (0.429) | (0.429) | (0.429) | (0.429) | (0.429) |
| Two | 0.170 | 0.171 | 0.161 | 0.168 | 0.168 |
|  | (0.405) | (0.405) | (0.405) | (0.405) | (0.406) |
| More than 2 | 0.231 | 0.231 | 0.223 | 0.229 | 0.230 |
|  | (0.398) | (0.398) | (0.398) | (0.398) | (0.398) |
| Missing flag | 0.297 | 0.294 | 0.291 | 0.298 | 0.296 |
|  | (0.598) | (0.599) | (0.598) | (0.599) | (0.599) |
| Number of bathrooms (room with a bath/shower or both) (Ref: none) |  |  |  |  |  |
| One | -0.782 | -0.781 | -0.798 | -0.789 | -0.789 |
|  | (0.815) | (0.813) | (0.819) | (0.815) | (0.816) |
| Two | -0.986 | -0.985 | -1.006 | -0.995 | -0.995 |
|  | (0.821) | (0.819) | (0.825) | (0.821) | (0.822) |
| More than 2 | -0.991 | -0.989 | -1.008 | -1.000 | -0.998 |
|  | (0.823) | (0.822) | (0.827) | (0.823) | (0.824) |
| Missing flag | -0.823 | -0.818 | -0.834 | -0.822 | -0.821 |
|  | (1.027) | (1.025) | (1.030) | (1.027) | (1.027) |
| Availability of dishwasher at home (Ref: no) |  |  |  |  |  |
| Yes | -0.460*** | -0.460*** | -0.460*** | -0.459*** | -0.459*** |
|  | (0.100) | (0.100) | (0.100) | (0.100) | (0.100) |
| Missing flag | -0.230 | -0.228 | -0.220 | -0.233 | -0.226 |
|  | (0.688) | (0.687) | (0.686) | (0.687) | (0.686) |
| Number of times you and your family travel out of Spain for a holiday / vacation last year (Ref: Not at all) |  |  |  |  |  |
| Once | -0.278*** | -0.279*** | -0.279*** | -0.280*** | -0.280*** |
|  | (0.094) | (0.094) | (0.094) | (0.094) | (0.094) |
| Twice | -0.576*** | -0.577*** | -0.573*** | -0.576*** | -0.575*** |
|  | (0.130) | (0.130) | (0.130) | (0.130) | (0.130) |
| More than twice | -0.538*** | -0.540*** | -0.534*** | -0.537*** | -0.535*** |
|  | (0.155) | (0.155) | (0.155) | (0.155) | (0.155) |
| Missing flag | 0.515 | 0.511 | 0.505 | 0.504 | 0.503 |
|  | (0.715) | (0.715) | (0.711) | (0.713) | (0.712) |
| Value the well-off of the family (Ref: not at all) |  |  |  |  |  |
| Not very well-off | -0.772 | -0.768 | -0.776 | -0.776 | -0.774 |
|  | (0.612) | (0.611) | (0.612) | (0.611) | (0.612) |
| Average | -1.539*** | -1.536*** | -1.543*** | -1.543*** | -1.542*** |
|  | (0.578) | (0.577) | (0.578) | (0.577) | (0.578) |
| Quite well-off | -1.757*** | -1.755*** | -1.754*** | -1.760*** | -1.757*** |
|  | (0.609) | (0.608) | (0.608) | (0.608) | (0.608) |
| Very well-off | -1.840*** | -1.837*** | -1.838*** | -1.841*** | -1.839*** |
|  | (0.622) | (0.621) | (0.621) | (0.621) | (0.621) |
| Missing flag | -1.874*** | -1.875*** | -1.878*** | -1.882*** | -1.879*** |
|  | (0.637) | (0.636) | (0.637) | (0.635) | (0.636) |
| Frequency of doing vigorous physical activity (Ref: never) |  |  |  |  |  |
| Less than once a month | -0.004 | -0.005 | -0.011 | -0.005 | -0.007 |
|  | (0.225) | (0.226) | (0.225) | (0.226) | (0.225) |
| Once a month | 0.228 | 0.226 | 0.232 | 0.229 | 0.229 |
|  | (0.245) | (0.245) | (0.245) | (0.245) | (0.245) |
| Once a week | 0.416** | 0.416** | 0.416** | 0.419** | 0.417** |
|  | (0.194) | (0.195) | (0.195) | (0.194) | (0.194) |
| 2-3 times a week | 0.186 | 0.185 | 0.182 | 0.186 | 0.183 |
|  | (0.185) | (0.185) | (0.185) | (0.185) | (0.185) |
| 4-6 times a week | 0.092 | 0.092 | 0.089 | 0.094 | 0.091 |
|  | (0.203) | (0.203) | (0.204) | (0.203) | (0.203) |
| Every day | -0.302 | -0.304 | -0.305 | -0.304 | -0.304 |
|  | (0.195) | (0.195) | (0.195) | (0.195) | (0.195) |
| Missing flag | -0.103 | -0.105 | -0.098 | -0.101 | -0.102 |
|  | (0.300) | (0.300) | (0.300) | (0.299) | (0.300) |
| Exercise - hours a week (Ref: none) |  |  |  |  |  |
| Half an hour | 0.172 | 0.171 | 0.169 | 0.171 | 0.171 |
|  | (0.152) | (0.152) | (0.152) | (0.151) | (0.152) |
| 1 hour | 0.232* | 0.232* | 0.230* | 0.231* | 0.232* |
|  | (0.130) | (0.130) | (0.130) | (0.130) | (0.130) |
| 2-3 hours | 0.175 | 0.175 | 0.174 | 0.175 | 0.175 |
|  | (0.128) | (0.128) | (0.128) | (0.128) | (0.128) |
| 4-6 hours | 0.364** | 0.363** | 0.360** | 0.362** | 0.361** |
|  | (0.155) | (0.155) | (0.155) | (0.155) | (0.155) |
| 7 hours or more | 0.155 | 0.157 | 0.157 | 0.158 | 0.158 |
|  | (0.163) | (0.163) | (0.162) | (0.163) | (0.163) |
| Missing flag | 0.314 | 0.315 | 0.308 | 0.313 | 0.312 |
|  | (0.301) | (0.301) | (0.301) | (0.300) | (0.301) |
| Constant | 22.187*** | 22.182*** | 22.221*** | 22.201*** | 22.200*** |
|  | (1.091) | (1.090) | (1.094) | (1.091) | (1.093) |
|  |  |  |  |  |  |
| Observations | 6,064 | 8,461 | 8,516 | 8,380 | 8,355 |
| *F statistic* of Stock and Yogo (2005) test of weak instruments | 19.026*** | 31.282*** | 2.555 | 6.665** | 10.308*** |

Dependent variable: ordinal dependent variables in Equation (2) and Body Mass Index (continuous variable) in Equation (3).

Method of estimation: conditional (recursive) mixed process estimator. Ordered probit model employed in Equation (2) and OLS in Equation (3). Standard errors are in parentheses and are clustered at school level. In equation (2), coefficients referred to probit coefficients. Full estimates of marginal effects are available upon request to authors.

The null hypothesis of the Stock and Yogo test (2005) is that the set of instrument is weak.

Coefficient: ***significant at 1%, ** at 5%, * at 10%.

Source: Authors’ own calculations.

Table A6. Full set of parameter estimates of peers’ support outcomes

| **Equation (2). Peers support outcomes** | | | | |
| --- | --- | --- | --- | --- |
|  | **Friends try to help** | **Can count on friends** | **Friends to share joys with** | **Can talk about problems with**  **friends** |
| BMI | -0.108*** | -0.076*** | 0.007 | 0.032 |
|  | (0.028) | (0.027) | (0.031) | (0.029) |
| Female (Ref: Male) | 0.278*** | 0.280*** | 0.497*** | 0.394*** |
|  | (0.032) | (0.030) | (0.029) | (0.028) |
| Immigrant status (Ref: Native) |  |  |  |  |
| First generation | -0.164*** | -0.148*** | -0.190*** | -0.204*** |
|  | (0.046) | (0.041) | (0.040) | (0.041) |
| Second generation | -0.116*** | -0.100*** | -0.070* | -0.087** |
|  | (0.037) | (0.038) | (0.039) | (0.040) |
| Father occupation SES (Ref: Low) |  |  |  |  |
| Medium-Low | 0.009 | -0.011 | -0.033 | -0.028 |
|  | (0.039) | (0.040) | (0.040) | (0.041) |
| Medium | -0.045 | -0.028 | -0.044 | -0.039 |
|  | (0.066) | (0.067) | (0.070) | (0.070) |
| Medium-High | 0.012 | 0.010 | 0.035 | 0.042 |
|  | (0.053) | (0.051) | (0.055) | (0.056) |
| High | 0.023 | -0.030 | 0.012 | 0.038 |
|  | (0.053) | (0.048) | (0.053) | (0.050) |
| Unclassifiable | -0.003 | -0.044 | -0.035 | -0.054 |
|  | (0.039) | (0.038) | (0.040) | (0.039) |
| Mother occupation SES (Ref: Low) |  |  |  |  |
| Medium-Low | 0.007 | 0.032 | 0.062 | 0.047 |
|  | (0.044) | (0.047) | (0.051) | (0.050) |
| Medium | 0.012 | 0.033 | 0.027 | 0.032 |
|  | (0.044) | (0.046) | (0.051 | (0.051) |
| Medium-High | 0.085 | 0.082 | 0.121* | 0.104 |
|  | (0.056) | (0.062) | (0.066) | (0.065) |
| High | 0.039 | 0.095* | 0.081 | 0.077 |
|  | (0.049) | (0.052) | (0.056) | (0.054) |
| Unclassifiable | 0.001 | 0.041 | -0.003 | 0.019 |
|  | (0.037) | (0.041) | (0.043) | (0.042) |
| Family own a car, van or truck (Ref: none) |  |  |  |  |
| One | 0.063 | 0.032 | -0.046 | -0.028 |
|  | (0.091) | (0.087) | (0.093) | (0.094) |
| Two or more | 0.127 | 0.104 | -0.045 | -0.007 |
|  | (0.093) | (0.088) | (0.094) | (0.095) |
| Missing flag | 0.373 | 0.376 | 0.196 | 0.147 |
|  | (0.234) | (0.227) | (0.258) | (0.229) |
| Own bedroom (Ref: No) |  |  |  |  |
| Yes | -0.006 | -0.032 | -0.037 | 0.014 |
|  | (0.035) | (0.035) | (0.038) | (0.036) |
| Missing flag | -0.108 | 0.147 | 0.196 | 0.303* |
|  | (0.158) | (0.176) | (0.157) | (0.161) |
| Number of computers family owned (including laptops and tablets, not including game consoles and smartphones) (Ref: none) |  |  |  |  |
| One | 0.152 | 0.135 | 0.050 | 0.144 |
|  | (0.116) | (0.126) | (0.118) | (0.128) |
| Two | 0.243** | 0.221* | 0.144 | 0.205 |
|  | (0.120) | (0.123) | (0.119) | (0.132) |
| More than 2 | 0.227* | 0.211* | 0.125 | 0.183 |
|  | (0.120) | (0.122) | (0.118) | (0.129) |
| Missing flag | 0.291 | 0.147 | -0.014 | 0.015 |
|  | (0.198) | (0.198) | (0.186) | (0.197) |
| Number of bathrooms (room with a bath/shower or both) (Ref: none) |  |  |  |  |
| One | 0.139 | 0.393** | 0.594*** | 0.399** |
|  | (0.207) | (0.187) | (0.152) | (0.177) |
| Two | 0.121 | 0.337* | 0.574*** | 0.389** |
|  | (0.210) | (0.187) | (0.153) | (0.177) |
| More than 2 | 0.030 | 0.264 | 0.496*** | 0.311* |
|  | (0.211) | (0.190) | (0.158) | (0.182) |
| Missing flag | 0.159 | 0.449 | 1.055*** | 0.699** |
|  | (0.355) | (0.348) | (0.369) | (0.352) |
| Availability of dishwasher at home (Ref: no) |  |  |  |  |
| Yes | 0.030 | 0.042 | 0.085** | 0.101*** |
|  | (0.038) | (0.037) | (0.039) | (0.039) |
| Missing flag | 0.110 | -0.129 | -0.317 | -0.183 |
|  | (0.226) | (0.206) | (0.243) | (0.203) |
| Number of times you and your family travel out of Spain for a holiday / vacation last year (Ref: Not at all) |  |  |  |  |
| Once | 0.050 | 0.029 | 0.086** | 0.108*** |
|  | (0.035) | (0.035) | (0.039) | (0.038) |
| Twice | 0.039 | 0.030 | 0.086 | 0.109** |
|  | (0.053) | (0.055) | (0.053) | (0.053) |
| More than twice | 0.003 | -0.015 | 0.036 | 0.027 |
|  | (0.056) | (0.056) | (0.059) | (0.058) |
| Missing flag | -0.343 | -0.327 | -0.352 | -0.291 |
|  | (0.236) | (0.269) | (0.253) | (0.229) |
| Value the well-off of the family (Ref: not at all) |  |  |  |  |
| Not very well-off | 0.152 | 0.172 | 0.192 | 0.208 |
|  | (0.147) | (0.136) | (0.155) | (0.146) |
| Average | 0.277* | 0.238* | 0.316** | 0.337** |
|  | (0.146) | (0.135) | (0.155) | (0.142) |
| Quite well-off | 0.173 | 0.160 | 0.280* | 0.357** |
|  | (0.152) | (0.145) | (0.163) | (0.152) |
| Very well-off | 0.119 | 0.042 | 0.094 | 0.260 |
|  | (0.187) | (0.174) | (0.189) | (0.178) |
| Missing flag | 0.331* | 0.191 | 0.330* | 0.323* |
|  | (0.190) | (0.180) | (0.197) | (0.186) |
| Frequency of doing vigorous physical activity (Ref: never) |  |  |  |  |
| Less than once a month | -0.106 | -0.083 | -0.053 | -0.024 |
|  | (0.072) | (0.074) | (0.075) | (0.071) |
| Once a month | -0.060 | -0.074 | -0.108 | -0.094 |
|  | (0.084) | (0.082) | (0.088) | (0.084) |
| Once a week | 0.018 | -0.046 | -0.031 | -0.064 |
|  | (0.066) | (0.067) | (0.068) | (0.066) |
| 2-3 times a week | 0.055 | 0.008 | 0.021 | -0.015 |
|  | (0.062) | (0.064) | (0.066) | (0.062) |
| 4-6 times a week | 0.030 | 0.012 | 0.028 | 0.003 |
|  | (0.067) | (0.065) | (0.071) | (0.066) |
| Every day | 0.195*** | 0.134** | 0.206*** | 0.182*** |
|  | (0.071) | (0.067) | (0.075) | (0.069) |
| Missing flag | 0.055 | -0.136 | -0.107 | -0.088 |
|  | (0.128) | (0.131) | (0.130) | (0.122) |
| Exercise - hours a week (Ref: none) |  |  |  |  |
| Half an hour | 0.009 | -0.021 | -0.046 | -0.016 |
|  | (0.059) | (0.061) | (0.059) | (0.058) |
| 1 hour | 0.064 | 0.079 | 0.054 | 0.056 |
|  | (0.052) | (0.052) | (0.056) | (0.054) |
| 2-3 hours | 0.136*** | 0.143*** | 0.119** | 0.152*** |
|  | (0.049) | (0.050) | (0.057) | (0.053) |
| 4-6 hours | 0.118** | 0.134** | 0.155** | 0.139** |
|  | (0.059) | (0.060) | (0.065) | (0.060) |
| 7 hours or more | 0.154** | 0.151** | 0.199*** | 0.211*** |
|  | (0.064) | (0.062) | (0.063) | (0.064) |
| Missing flag | 0.015 | 0.068 | -0.067 | 0.025 |
|  | (0.127) | (0.126) | (0.128) | (0.116) |
| Constant cut1 | -2.881*** | -2.141*** | -0.159 | 0.343 |
|  | (0.688) | (0.621) | (0.761) | (0.716) |
| Constant cut2 | -2.600*** | -1.836*** | 0.091 | 0.593 |
|  | (0.695) | (0.628) | (0.759) | (0.713) |
| Constant cut3 | -2.301*** | -1.565** | 0.300 | 0.822 |
|  | (0.702) | (0.631) | (0.759) | (0.710) |
| Constant cut4 | -1.912*** | -1.207* | 0.616 | 1.144 |
|  | (0.712) | (0.637) | (0.757) | (0.706) |
| Constant cut5 | -1.508** | -0.843 | 0.939 | 1.464** |
|  | (0.722) | (0.641) | (0.753) | (0.703) |
| Constant cut6 | -0.973 | -0.290 | 1.424* | 1.958*** |
|  | (0.735) | (0.648) | (0.750) | (0.695) |
| **Equation (3). BMI equation** | | | | |
| Alcohol consumption (Ref: Never) |  |  |  |  |
| 1-2 days | 0.975*** | 0.978*** | 0.982*** | 0.987*** |
|  | (0.112) | (0.110) | (0.108) | (0.107) |
| 3-5 days | 1.101*** | 1.108*** | 1.103*** | 1.084*** |
|  | (0.177) | (0.181) | (0.184) | (0.187) |
| 6-9 days | 0.879*** | 0.926*** | 0.972*** | 0.981*** |
|  | (0.227) | (0.222) | (0.235) | (0.233) |
| 10-19 days | 1.973*** | 1.931*** | 1.860*** | 1.859*** |
|  | (0.464) | (0.481) | (0.498) | (0.490) |
| 20-29 days | 1.778* | 1.830* | 1.880* | 1.921** |
|  | (0.971) | (0.989) | (0.977) | (0.956) |
| 30 days (or more) | 0.265 | 0.347 | 0.461 | 0.491 |
|  | (0.371) | (0.385) | (0.385) | (0.377) |
| Missing flag | 0.514*** | 0.469*** | 0.461** | 0.459** |
|  | (0.175) | (0.180) | (0.186) | (0.183) |
| Female (Ref: Male) | -0.344*** | -0.344*** | -0.344*** | -0.344*** |
|  | (0.075) | (0.075) | (0.075) | (0.075) |
| Immigrant status (Ref: Native) |  |  |  |  |
| First generation | 0.308** | 0.310** | 0.310** | 0.310** |
|  | (0.144) | (0.144) | (0.144) | (0.144) |
| Second generation | -0.055 | -0.055 | -0.054 | -0.054 |
|  | (0.118) | (0.118) | (0.118) | (0.118) |
| Father occupation SES (Ref: Low) |  |  |  |  |
| Medium-Low | -0.116 | -0.117 | -0.118 | -0.118 |
|  | (0.106) | (0.107) | (0.107) | (0.107) |
| Medium | -0.151 | -0.151 | -0.151 | -0.151 |
|  | (0.183) | (0.183) | (0.183) | (0.183) |
| Medium-High | -0.084 | -0.084 | -0.085 | -0.085 |
|  | (0.148) | (0.148) | (0.148) | (0.148) |
| High | -0.467*** | -0.468*** | -0.469*** | -0.469*** |
|  | (0.124) | (0.124) | (0.124) | (0.124) |
| Unclassifiable | 0.013 | 0.012 | 0.012 | 0.012 |
|  | (0.109) | (0.109) | (0.109) | (0.109) |
| Mother occupation SES (Ref: Low) |  |  |  |  |
| Medium-Low | -0.369*** | -0.368*** | -0.367*** | -0.367*** |
|  | (0.143) | (0.143) | (0.143) | (0.143) |
| Medium | -0.310** | -0.309** | -0.308** | -0.308** |
|  | (0.130) | (0.131) | (0.130) | (0.130) |
| Medium-High | -0.502*** | -0.502*** | -0.502*** | -0.503*** |
|  | (0.177) | (0.177) | (0.177) | (0.177) |
| High | -0.531*** | -0.531*** | -0.530*** | -0.530*** |
|  | (0.139) | (0.139) | (0.139) | (0.139) |
| Unclassifiable | -0.235** | -0.234** | -0.233** | -0.233* |
|  | (0.119) | (0.119) | (0.119) | (0.119) |
| Family own a car, van or truck (Ref: none) |  |  |  |  |
| One | 0.316 | 0.315 | 0.315 | 0.316 |
|  | (0.245) | (0.245) | (0.245) | (0.245) |
| Two or more | 0.388 | 0.388 | 0.387 | 0.388 |
|  | (0.255) | (0.255) | (0.255) | (0.255) |
| Missing flag | -0.094 | -0.093 | -0.091 | -0.091 |
|  | (0.602) | (0.602) | (0.602) | (0.602) |
| Own bedroom (Ref: No) |  |  |  |  |
| Yes | 0.204* | 0.202* | 0.202* | 0.202* |
|  | (0.117) | (0.117) | (0.117) | (0.117) |
| Missing flag | -0.545* | -0.549* | -0.551* | -0.551* |
|  | (0.326) | (0.325) | (0.324) | (0.324) |
| Number of computers family owned (including laptops and tablets, not including game consoles and smartphones) (Ref: none) |  |  |  |  |
| One | 0.155 | 0.154 | 0.153 | 0.152 |
|  | (0.429) | (0.429) | (0.429) | (0.429) |
| Two | 0.167 | 0.165 | 0.164 | 0.163 |
|  | (0.405) | (0.405) | (0.405) | (0.405) |
| More than 2 | 0.228 | 0.227 | 0.226 | 0.225 |
|  | (0.398) | (0.398) | (0.398) | (0.398) |
| Missing flag | 0.292 | 0.292 | 0.293 | 0.292 |
|  | (0.599) | (0.599) | (0.599) | (0.598) |
| Number of bathrooms (room with a bath/shower or both) (Ref: none) |  |  |  |  |
| One | -0.790 | -0.793 | -0.794 | -0.794 |
|  | (0.816) | (0.817) | (0.817) | (0.817) |
| Two | -0.999 | -1.002 | -1.002 | -1.002 |
|  | (0.822) | (0.823) | (0.823) | (0.823) |
| More than 2 | -1.001 | -1.004 | -1.004 | -1.004 |
|  | (0.824) | (0.826) | (0.826) | (0.826) |
| Missing flag | -0.817 | -0.823 | -0.827 | -0.830 |
|  | (1.027) | (1.028) | (1.028) | (1.028) |
| Availability of dishwasher at home (Ref: no) |  |  |  |  |
| Yes | -0.459*** | -0.459*** | -0.460*** | -0.460*** |
|  | (0.100) | (0.100) | (0.100) | (0.100) |
| Missing flag | -0.219 | -0.220 | -0.221 | -0.221 |
|  | (0.686) | (0.685) | (0.685) | (0.686) |
| Number of times you and your family travel out of Spain for a holiday / vacation last year (Ref: Not at all) |  |  |  |  |
| Once | -0.281*** | -0.281*** | -0.280*** | -0.280*** |
|  | (0.094) | (0.094) | (0.094) | (0.094) |
| Twice | -0.575*** | -0.574*** | -0.574*** | -0.574*** |
|  | (0.130) | (0.130) | (0.130) | (0.130) |
| More than twice | -0.536*** | -0.535*** | -0.535*** | -0.535*** |
|  | (0.155) | (0.155) | (0.155) | (0.155) |
| Missing flag | 0.494 | 0.498 | 0.502 | 0.504 |
|  | (0.712) | (0.711) | (0.712) | (0.712) |
| Value the well-off of the family (Ref: not at all) |  |  |  |  |
| Not very well-off | -0.775 | -0.776 | -0.776 | -0.775 |
|  | (0.612) | (0.612) | (0.612) | (0.611) |
| Average | -1.543*** | -1.544*** | -1.543*** | -1.542*** |
|  | (0.578) | (0.578) | (0.578) | (0.578) |
| Quite well-off | -1.755*** | -1.755*** | -1.755*** | -1.754*** |
|  | (0.608) | (0.608) | (0.608) | (0.608) |
| Very well-off | -1.837*** | -1.837*** | -1.838*** | -1.837*** |
|  | (0.621) | (0.621) | (0.621) | (0.621) |
| Missing flag | -1.882*** | -1.881*** | -1.879*** | -1.878*** |
|  | (0.636) | (0.636) | (0.637) | (0.637) |
| Frequency of doing vigorous physical activity (Ref: never) |  |  |  |  |
| Less than once a month | -0.011 | -0.011 | -0.010 | -0.010 |
|  | (0.226) | (0.226) | (0.225) | (0.225) |
| Once a month | 0.229 | 0.230 | 0.231 | 0.231 |
|  | (0.245) | (0.245) | (0.245) | (0.245) |
| Once a week | 0.416** | 0.416** | 0.416** | 0.416** |
|  | (0.195) | (0.195) | (0.195) | (0.195) |
| 2-3 times a week | 0.181 | 0.182 | 0.183 | 0.183 |
|  | (0.185) | (0.185) | (0.185) | (0.185) |
| 4-6 times a week | 0.091 | 0.090 | 0.090 | 0.090 |
|  | (0.204) | (0.203) | (0.203) | (0.203) |
| Every day | -0.307 | -0.306 | -0.305 | -0.305 |
|  | (0.195) | (0.195) | (0.195) | (0.195) |
| Missing flag | -0.102 | -0.100 | -0.100 | -0.100 |
|  | (0.299) | (0.299) | (0.299) | (0.300) |
| Exercise - hours a week (Ref: none) |  |  |  |  |
| Half an hour | 0.169 | 0.169 | 0.169 | 0.169 |
|  | (0.152) | (0.152) | (0.152) | (0.152) |
| 1 hour | 0.232* | 0.231* | 0.231* | 0.231* |
|  | (0.130) | (0.130) | (0.130) | (0.130) |
| 2-3 hours | 0.174 | 0.174 | 0.174 | 0.174 |
|  | (0.128) | (0.128) | (0.128) | (0.128) |
| 4-6 hours | 0.360** | 0.360** | 0.360** | 0.360** |
|  | (0.155) | (0.155) | (0.155) | (0.155) |
| 7 hours or more | 0.160 | 0.159 | 0.157 | 0.157 |
|  | (0.163) | (0.163) | (0.162) | (0.162) |
| Missing flag | 0.311 | 0.310 | 0.310 | 0.310 |
|  | (0.301) | (0.301) | (0.301) | (0.301) |
| Constant | 22.208*** | 22.214*** | 22.214*** | 22.214*** |
|  | (1.093) | (1.094) | (1.093) | (1.093) |
|  |  |  |  |  |
| Observations | 8,693 | 8,676 | 8,659 | 8,626 |
| *F statistic* of Stock and Yogo (2005) test of weak instruments | 4.559** | 0.610 | 2.722* | 7.174*** |

Dependent variable: ordinal dependent variables in Equation (2) and Body Mass Index (continuous variable) in Equation (3).

Method of estimation: conditional (recursive) mixed process estimator. Ordered probit model employed in Equation (2) and OLS in Equation (3). Standard errors are in parentheses and are clustered at school level. In equation (2), coefficients referred to probit coefficients. Full estimates of marginal effects are available upon request to authors.

The null hypothesis of the Stock and Yogo test (2005) is that the set of instrument is weak.

Coefficient: ***significant at 1%, ** at 5%, * at 10%.

Source: Authors’ own calculations.
