# Supplementary material for: Influence of body mass index on health complains and life satisfaction
Source: Qual Life Res. 2023 Dec 1;33(3):705–19. doi: 10.1007/s11136-023-03557-0 (PMC10894113; doi:10.1007/s11136-023-03557-0)
Supplement: Supplementary file 2 — Supplementary file2 (DOCX 17 kb) [file 11136_2023_3557_MOESM2_ESM.docx]

**Appendix B**

The instrumental variable analysis is increasingly used for learning about causality using observational data (Swanson & Hernán, 2018). This approach is employed to address endogeneity, which arises when unobservable factors (u) in the model are correlated with the treatment variable ($BMI$) ‒ $Cov\left( {BMI}_{i}, u_{i} \right)\neq0$. This correlation can result in biased estimates of the effect of the treatment (BMI) on the outcome ($O$*) on equation (1).

To ascertain the causal effect of the treatment on the outcome, the instrument (Z) must exhibit a correlation with the endogenous variable ‒ denoted as $Cov\left( {BMI}_{i}, Z_{i} \right)\neq0$‒, and it should be exogenous ‒ meaning it is not correlated with the error term $Cov\left( Z_{i}, u_{i} \right)=0$. In specific terms, valid instruments must meet the following conditions (see Angrist & Pischke, 2009; Wooldridge, 2010, for algebraic and formal definitions of the assumptions):

1. The *relevance condition* or *first stage*. This condition indicates that the instrument should be strongly associated with the treatment. The Stock and Yogo test, also known as the Cragg-Donald Wald F-statistic, is a statistical test used to assess the validity of instrumental variables. Specifically, it helps determine whether the instrumental variable is correlated with the endogenous variable, which is a key requirement for instrumental variable analysis. The null hypothesis is that the IV is weakly correlated with the instrument. If it fails to reject the null hypothesis, the instrument is weak and the results of the IV estimations may be unreliable. Besides having literature supports of an existing association between alcohol consumption and overweight (Traversy & Chaput, 2015), we will observe in the estimations that the frequency of alcohol consumption is clearly correlated with body mass index, conditional on other covariates. We will also see that the Stock and Yogo (2005) test of weak instruments rejects the null hypothesis (i.e. the instrument is weak) and confirms that the relevance condition holds^[[1]](#footnote-1)^.
2. The *independence*/*exogeneity assumption*. This condition stablishes that the instrument is randomly assigned or “as good as randomly assigned” meaning that it is uncorrelated to the omitted variables we might like to control for. This entails that the instrument cannot be correlated with the error term, that is $Cov\left( Z_{i}, u_{i} \right)=0$. After including the control variables, alcohol consumption is assumed to be as good as random, given that alcohol consumption in adolescence may be mostly driven by socioeconomic and cultural variables, which are thoroughly controlled in the model with father and mother socioeconomic status and home possessions.
3. The *exclusion restriction*. This condition stablishes that the instrumental variable may not have an influence on the dependent variable through any other channel except its impact on the endogenous variable. It means that there is a sole channel for the influence of the instrument on the outcomes, that is the endogenous variable. According to this condition, the alcohol consumption may exert their influence on the outcomes variable solely through the ${BMI}_{i}.$ We argue that this assumption may hold based on the fact that other plausible channels of influence have been controlled for.
4. The *monotonicity property*. While the instrument may have no effect on some individuals, all of those who are affected should be affected unidirectionally. It means that the instrument cannot increase the treatment level for some individuals and decrease it for others. This condition means that those individuals who drank alcohol in the last 30 days and their BMI is influenced by it would see this in one direction, i.e. BMI will increase (as highlighted by Kleiner et al., 2004; Traversy & Chaput, 2015). As we will also see in the first stage estimations in the Results section, higher alcohol consumption is associated with higher BMI.

1. The null hypothesis (the instrument is weak) cannot be rejected in the modelling of two variables: “been bullied” (Table A5, Appendix) and “can count on friends” (Table A6, Appendix). [↑](#footnote-ref-1)
